# Rice Calcineurin B-Like Protein-Interacting Protein Kinase 31 (OsCIPK31) Is Involved in the Development of Panicle Apical Spikelets

**DOI:** 10.3389/fpls.2018.01661

**Published:** 2018-11-19

**Authors:** Yongbin Peng, Feixue Hou, Que Bai, Peizhou Xu, Yongxiang Liao, Hongyu Zhang, Chaojian Gu, Xiaoshu Deng, Tingkai Wu, Xiaoqiong Chen, Asif Ali, Xianjun Wu

**Affiliations:** Rice Research Institute, Sichuan Agricultural University at Wenjiang, Chengdu, China

**Keywords:** panicle apical abortion, CIPK, apical dominance, IAA, auxin, MAPK signaling, ROS

## Abstract

Panicle apical abortion (PAA) causes severe yield losses in rice production, but details about its development and molecular basis remain elusive. Herein, a PAA mutant, *paa1019*, was identified among the progeny of an elite indica maintainer rice line Yixiang 1B (YXB) mutagenized population obtained using ethyl methyl sulfonate. The abortion rate of spikelets in *paa1019* was observed up to 60%. Genetic mapping combined with Mutmap analysis revealed that *LOC_Os03g20380* harbored a single-bp substitution (C to T) that altered its transcript length. This gene encodes calcineurin B-like protein-interacting protein kinase 31 (OsCIPK31) localized into the cytoplasm, and is preferentially expressed in transport tissues of rice. Complementation of *paa1019* by transferring the open reading frame of *LOC_Os03g20380* from YXB reversed the mutant phenotype, and conversely, gene editing by knocking out of *OsCIPK31* in YXB results in PAA phenotype. Our results support that *OsCIPK31* plays an important role in panicle development. We found that dysregulation is caused by the disruption of *OsCIPK31* function due to excessive accumulation of ROS, which ultimately leads to cell death in rice panicle. *OsCIPK31* and MAPK pathway might have a synergistic effect to lead ROS accumulation in response to stresses. Meanwhile the PAA distribution is related to IAA hormone accumulation in the panicle. Our study provides an understanding of the role of OsCIPK31 in panicle development by responding to various stresses and phytohormones.

## Introduction

Plant architectural features (Wang and Li, 2008), especially panicle structure, size, and shape (Sakamoto and Matsuoka, 2004), are the major factors influencing grain yield, and improving panicle architecture is an important goal in rice (*Oryza sativa*) breeding programs (Sakamoto and Matsuoka, 2008). During panicle development, the inflorescence meristem initiates primary branch meristems, which also produce several secondary branch meristems, and ultimately, primary panicle branches (PBs) and secondary panicle branches (SBs) differentiate into spikelets. Finally, the inflorescence undergoes a long period of branch elongation and spikelet development before heading (Li et al., 2009).

Many regulatory genes are involved in the determination of panicle architecture. The pattern of axillary meristem (AM) formation is of special significance and it determines the complexity of the whole plant architecture. Transcription factors such as MONOCULM1 (*MOC1*) (Li et al., 2003), LAX1 (Komatsu et al., 2003; Oikawa and Kyozuka, 2009), *LAX2* (Tabuchi et al., 2011), and frizzy panicle (FZP) (Komatsu et al., 2001) regulate the pattern formation of AMs, whereas SHORT PANICLE1 (*SP1*) (Li et al., 2009) and ERECT PANICLE2 (*EP2*) (Zhu et al., 2010) regulate AM formation by controlling the transportation of unknown substrates. Phytohormones are also thought to influence panicle architecture (Mcsteen, 2009; Lu et al., 2017). Generation and outgrowth of lateral shoots are regulated by combined actions of hormones such as auxins, cytokinins, strigolactones, and environmental cues (Tabuchi et al., 2011). PBs and SBs are increased upon upregulation of CYTOKININ OXIDASE (*OsCKX2*) in the cytokinin synthesis pathway (Ashikari et al., 2005; Han et al., 2014), and are dependent on iterations of branching and spikelet meristem formation during the maintenance of determinacy and indeterminacy (Kellogg et al., 2013; Zhang and Yuan, 2014; Zhi et al., 2014). *RCN1* and *RCN2*, rice homologs of TERMINAL FLOWER/CENTRORADIALIS (*TFL*/*CEN*) that promote indeterminate inflorescence on branch and spikelet meristems in *Arabidopsis* and *Antirrhinum*, respectively (Bradley et al., 1996, 1997), lead to indeterminate inflorescence and a more branched panicle when ectopically expressed (Nakagawa et al., 2002). These indeterminacy inhibiting and promoting factors also interact with each other (Bradley et al., 1996).

In rice, panicle development can be roughly divided into two main stages; the early stage includes initiation of branch primordium and spikelet differentiation until panicle length remains short, whereas branch elongation and spikelet formation occur in the second stage (Li et al., 2009). Almost all characterized genes affecting panicle architecture participate in molecular activities during the first stage, and the mechanisms underlying branch elongation and spikelet development remain yet poorly understood. *SP1* negatively regulates panicle length, and *OsRAMOSA2 (RA2)* controls panicle architecture by regulating pedicel length, but also little is known about the control of terminal spikelet development.

Panicle apical abortion (PAA) is common in rice, and spikelet abortion can take place either in the apical or basal part of the panicle. Early stage degeneration of spikelets is one of the detrimental factors affecting the final grain yield (Senanayake et al., 1994; Ansari et al., 2003; Kobayasi and Imaki, 2008). PAA is commonly influenced by various environmental factors, but the underlying mechanisms and targets are poorly understood. Several quantitative trait loci (QTLs) have been reported for floret abortion that are located on different chromosomes (Yamagishi et al., 2004; Xu et al., 2007; Cheng et al., 2011; Tan et al., 2011), and the *SP1* gene influences the basal part of the panicle (Li et al., 2009). The *tut1* mutant displays abnormal development of anthers and pollen grains (Bai et al., 2015). The *PAA2* gene belongs to the NPF family and mutation in it leads to PAA phenotype in rice (Luo, 2016). Recently, *OsALMT7* is reported as a malate transporter that plays its role in the development of panicle apical portions (Heng et al., 2018). In addition, both the genetic and environmental factors can affect the degeneration of florets (González et al., 2011), which makes analysis more complicated. Herein, we report a novel rice mutant, *paa1019*, which is specifically defective in panicle development. Gene cloning and characterization indicate that *PAA1019* encodes OsCIPK31, a CIPK family kinase, which affects panicle development in rice. Possible roles of *CIPK31* in regulating the PAA phenotype are explored and discussed.

## Results

### Phenotype of the *paa1019* Mutant

**FIGURE 1:**
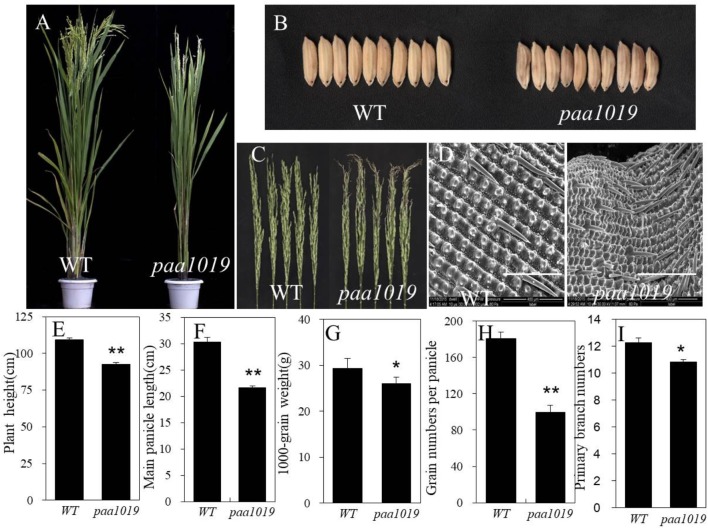
Phenotypes of the *paa1019* mutant. Phenotypes, microscopy analysis, and agronomic traits of wild-type (WT) and *paa1019* mutant plants. **(A)** Phenotype of the heading stage. **(B)** Phenotype of seeds. **(C)** Phenotype of panicles. **(D)** Microscopy analysis of the seed surface. **(E)** Quantification of plant height. **(F)** Main panicle length. **(G)** 1000-grain weight. **(H)**. Grain number per panicle. **(I)** Primary branch number. Statistical analysis was performed using Student’s *t*-tests. ^∗^*p* < 0.05, ^∗∗^*p* < 0.01. Scale bar in **(D)** = 400 μm.

Compared with wild-type (WT) YXB plants, *paa1019* exhibited a reduction in plant height (Figures 1A,E), longer growth duration (Supplementary Figure S1A), drastically reduced spikelet number per panicle, smaller main panicle length, reduced 1000-grain weight, and smaller erect panicles with clearly aborted tips (Figures 1F–H). Meanwhile, *paa1019* displayed the same number of tillers and secondary branches per panicle (Supplementary Figures S1B,C), and more primary branches per panicle (Figure 1I). Overall, unlike WT plants in which all spikelets were developed normally, the development of more than 60% of apical spikelets growth was arrested in *paa1019* (Figure 1C and Supplementary Figure S1D). Some areas of *paa1019* leaves lacked chlorophyll, and the net photosynthetic rate was lower than that in WT plants, whereas the carotene contents were higher (Supplementary Figures S2A–D). Furthermore, the malondialdehyde (MDA) contents of leaves in *paa1019* were higher (Supplementary Figure S2E), indicating peroxidation damage. After finishing grain filling, *paa1019* plants displayed brown lesions on glumes (Figure 1B). Electron microscopy (EM) revealed that grain filling in *paa1019* was incomplete compared with WT plants (Figure 1D).

### Isolation and Genetic Analysis of the *paa1019* Mutant

**FIGURE 2:**
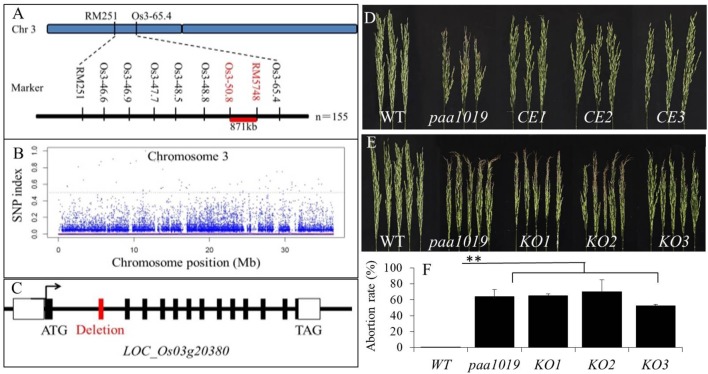
Genetic and physical maps of the *OsCIPK31* gene. **(A)** The *OsCIPK31* gene is located on chromosome 3 between Os3-50.8 and RM 5748. **(B)** Manhattan plot of chromosome 3. **(C)** Gene structure of *OsCIPK31*. The splicing pattern is altered in the mutant, resulting in the deletion of the second exon. **(D)** Phenotype of WT, *paa1019*, and complementation lines. *CE1 – CE3* represent the three complementation transgenic lines. **(E)** Phenotype of WT, *paa1019*, and knock-out lines. *KO1 – KO3* represent three knock-out transgenic lines. **(F)** Abortion rate analysis of lines corresponding to **(E)**. Statistical analysis was performed using Student’s *t-*tests, ^∗∗^*p* < 0.01.

Genetic analysis showed that *paa1019* was controlled by a single recessive nuclear locus with a segregation ratio of 3.0. To map the *PAA1019* locus, 550 pairs of simple sequence repeat (SSR) markers that are evenly distributed in the rice genome were selected to screen polymorphic markers between parent and mutant plants. The *OsPAA1019* locus was initially mapped to a region of 871 kb on chromosome 3 that is located between the markers Os3_50.8 and RM5748 (Figure 2A). Pooled DNA from 25 individuals displaying the PAA phenotype in F_2_ progeny was resequenced using the Illumina HiSeq 2500 platform. A sequence comparison showed several single-nucleotide polymorphisms (SNPs) between WT plants and the mutant pool. Fortunately, only one SNP (SNP-index = 100%) was found in the candidate region, which was localized in the junction between the first intron and the second exon of *LOC_Os03g20380* (Figure 2B). A single-base substitution (G to A) was detected at the position of 1101 nucleotide, and sequence comparison analysis revealed deletion of the entire second exon in *paa1019* (63 bp deletion; Figure 2C and Supplementary Figure S3). These results indicate that the PAA phenotype in the *paa1019* mutant likely arose from the single-base substitution in *LOC_Os03g20380*, and this gene encodes a CBL-interacting protein kinase, *OsCIPK31*, that belongs to plant CIPK family of proteins (Piao et al., 2010).

To perform complementation analysis, plasmid pBWA(V)BII containing the entire *LOC_Os03g20380* open reading frame (ORF) was introduced into the *paa1019* mutant. All the three resulting transgenic lines showed complementation of the *paa1019* phenotype (Figure 2D). Quantitative real-time RT-PCR (qRT-PCR) data revealed complementation lines expressed similar quantity of the *OsCIPK31* transcripts (Supplementary Figure S4). We also transformed a CRISPR-*Cas9* construct targeting the fourth exon of *OsCIPK31* into wild-type (YXB). Based on the phenotype of PAA, we identified and analyzed all the three independent transgenic lines (with frame shift; Supplementary Figure S5). To avoid the presence of off-targets, all the lines were backcrossed to the wild-type, respectively, and target lines were isolated from backcross populations. Strikingly, all the knock-out plants exhibited the apical panicle abortion phenotype (Figure 2E), with a degree of degradation degree comparable to the *paa1019* mutant (Figure 2F). The mode of mutation for these knock-out lines is shown in Supplementary Figure S5. Together, these results confirmed that *LOC_Os03g20380* indeed corresponds to the *PAA1019* gene.

### Spatiotemporal Analysis of Spikelet Degeneration Indicates Apical Dominance

**FIGURE 3:**
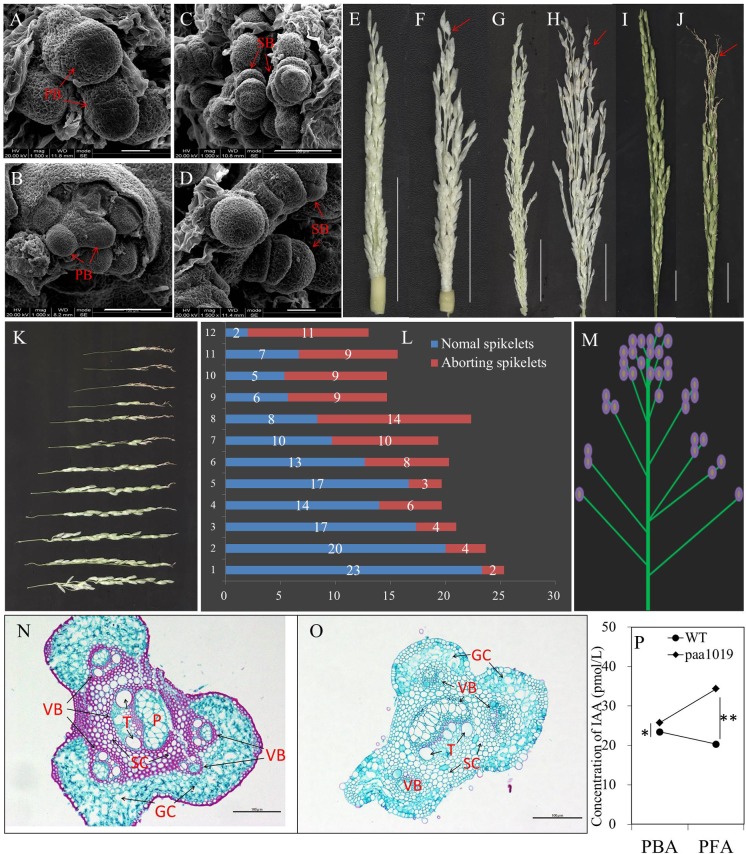
Characteristics of degraded spikelets. **(A–D)** Scanning electron microscopy (SEM) of shoot apical meristems (SAMs). Branch and spikelet primordia of WT **(A,C)** and *paa1019* **(B,D)** plants. PB, primary branches; SB, second branches. **(E–J)** Panicle development at different growth stages. Panicles were observed when the length was 4, 10, and 15 cm for WT **(E,G,I)** and *paa1019* **(F,H,J)** plants. **(K)** Phenotype of primary branches from the bottom to the top of the panicle in *paa1019*. **(L)** Statistics for the spikelet number correspond to **(K)**. **(M)** Model of apical abortion distribution in *paa1019*. **(N,O)** Transverse sections of pedicels. **(O)** Secondary pedicel in PAA. **(N)** Corresponding parts in WT plants. VB, vascular bundle; GC, green cells; SC, sclerenchymatous cells; T, vessel; P, phloem. **(P)** Concentration of indole-3-acetic acid (IAA/auxin). PBA, panicles before abortion; PFA, panicles following abortion. Scale bar = 100 μm **(A–D,N,O)** and 2 cm **(E–J)**. ^∗^*p* < 0.05, ^∗∗^*p* < 0.01.

To examine the developmental defects affecting spikelets, we carried out an in-depth analysis of panicle formation by comparing differences between WT and *paa1019* plants during representative developmental stages. Scanning Electron Microscopic (SEM) observations did not reveal significant morphological differences between *paa1019* and WT shoot apical meristems (SAMs) in vegetative or reproductive apices (Figures 3A–D). The *paa1019* plants initiated branch primordia and spikelet primordia in the similar way as WT plants, and the young panicle appeared to be normal in *paa1019* plants until they reached ∼4 cm in length. However, when the young panicle elongated gradually, the apical part of each primary branch in WT panicles elongated normally, whereas those in *paa1019* were significantly delayed or completely arrested, and the spikelets at the top of panicles were clearly found degenerated (Figures 3E–J). As panicle development proceeded, the degree of abortion was observed increasing in *paa1019* plants.

It is commonly believed that auxin accumulation in the apical meristem promotes terminal bud growth and inhibits lateral bud growth, and this is referred to as “apical dominance.” Differentiation of spikelet primordia on each primary branch is basipetal; development of apical spikelets always occurs earlier than basal spikelets. Consistently, the occurrence of PAA was consistent with previous studies (Chenjia et al., 2010). We also noticed that the degree of degradation was associated with spikelet location. First, the abnormal part appeared at the top of each primary branch (Figure 3K). Second, the rate of spikelet abortion in a single primary branch increased gradually from the bottom to the top of the panicle (Figure 3L); we observed only a few abnormal spikelets at the basal regions of panicles, whereas the upper regions barely showed normal development (Figure 3M). In addition, the content of indole-3-acetic acid (IAA/auxin) in the young panicles of *paa1019* plants was higher than in WT plants during panicle development (Figure 3P), indicating that IAA may be involved in regulating PAA. We examined transverse sections of pedicels, and found fewer vascular bundles and reduced green cells in *paa1019*. By contrast, regions of vascular bundles in pedicels were replaced with sclerenchymatous cells in WT plants (Figures 3N,O). Thus, a few structural changes appeared to cause blockage of nutrient transportation, leading to malnutrition during apical development.

### Reactive Oxygen Species (ROS) Accumulation Induces Cell Death in Panicle Apical Spikelets

**FIGURE 4:**
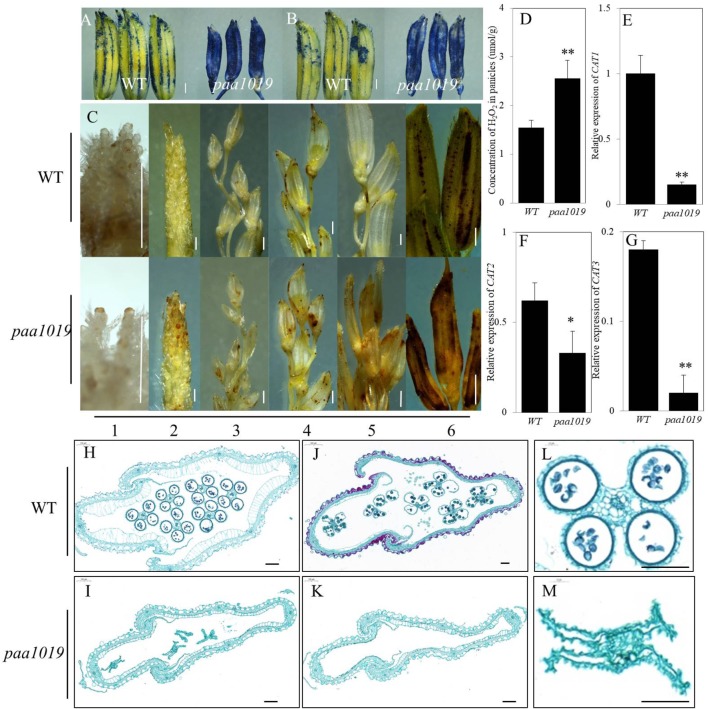
ROS accumulation leads to plasma membrane damage in *paa1019.* **(A)** Trypan blue staining. **(B)** Evans blue staining. **(C)** DAB staining at different growth stages. Primordium differentiation stage (1) = 20 days before flowering (DBF); (2) = 15 DBF; (3) = 10 DBF; (4) = 5 DBF; (5) = heading stage; (6) differences between WT and *paa1019*. **(D)** Concentration of H_2_O_2_ in panicles. **(E–G)** Relative expression of CAT1, CAT2, and CAT3 isozymes. Rice ACTIN was used as an internal control. Data are presented as mean ± SE (*n* = 3). **(H–M)** Transverse sections of spikelets at booting stages **(H,I)** and heading stages **(J,K)**. **(L,M)** Enlargement of stamens in **(H,I)**. Scale bar = 0.2 c **(C)** and 100 μm **(H–M)**. ^∗^*p* < 0.05, ^∗∗^*p* < 0.01.

To examine whether PAA was accompanied by programmed cell death (PCD), we analyzed PAA using trypan blue and Evans blue staining. Spikelets suffering PAA in *paa1019* displayed much darker blue staining (Figures 4A,B). ROS are well-known stress signaling molecules in both plants and animals, and excessive levels of H_2_O_2_ can damage the structure of cell walls, cell membranes, and macromolecules such as DNA and proteins (Gomez et al., 2004). To investigate the functions of ROS in rice panicle apical spikelet fate regulation, we first examined H_2_O_2_ distribution in apical spikelets using 3,3′-diaminobenzidine (DAB). H_2_O_2_ levels in apical spikelets in *paa1019* differed during different development stages (Figure 4C). We further confirmed the relative concentration of H_2_O_2_ in panicles in both WT and *paa1019* plants, and H_2_O_2_ increased in *paa1019* (Figure 4D). Catalase (CAT) is the key peroxidase acting in the biological defense system, and excessive H_2_O_2_ is converted by CATs into oxygen. *OsCAT1*, *OsCAT2*, and *OsCAT3* are CAT isozymes in rice. Consistent with our previous findings, all the three isozymes were expressed at lower levels in *paa1019* than WT plants (Figures 4E–G), indicating that ROS accumulation (H_2_O_2_) might affect the plasma membrane (PM) system, eventually leading to apical spikelet to cell death.

To further confirm damage caused by ROS accumulation, floral organ development was assessed during different stages before flowering. During the early stage of stamen and pistil development, between 20 and 15 days before flowering (DBF), there were no significant differences observed between WT and *paa1019* plants (Supplementary Figures S6A–D), but differences began to appear at 10 DBF and remained until maturity. Compared with WT, the androecium was withered and smaller, and trichomes attached to the gynoecium were reduced at 10 DBF in *paa1019* (Supplementary Figures S6E,F). Over time, the anther was filled with abundant pollen grains, and the gynoecium acquired numerous trichomes at 5 DBF in WT plants (Supplementary Figures S6G,H). By contrast, the corresponding parts were far less well developed in *paa1019*, and the anther and stigma were more withered, ovaries were fragile, and the filament and style offered minimal support. Although pollen grains were still visible in the pollen sac, I_2_-KI staining suggested these were not viable (Supplementary Figures S6K,L). All the reproductive organs were apoptotic in *paa1019* until flowering time (Supplementary Figures S6I,J). Moreover, to further understand the nature of spikelet abortion, we prepared cross-sections of spikelets along the middle region of the hull, which showed that the cell wall and cytoplasmic membrane of glumes and stamens were deformed during both booting and heading stages, consistent with the above observations (Figures 4H–M). These results suggest that that ROS accumulation indeed damages the PM structure of panicle apical spikelets, eventually leading to cell death.

### The *paa1019* Mutant Exhibits Hypersensitivity to Abiotic Stress

**FIGURE 5:**
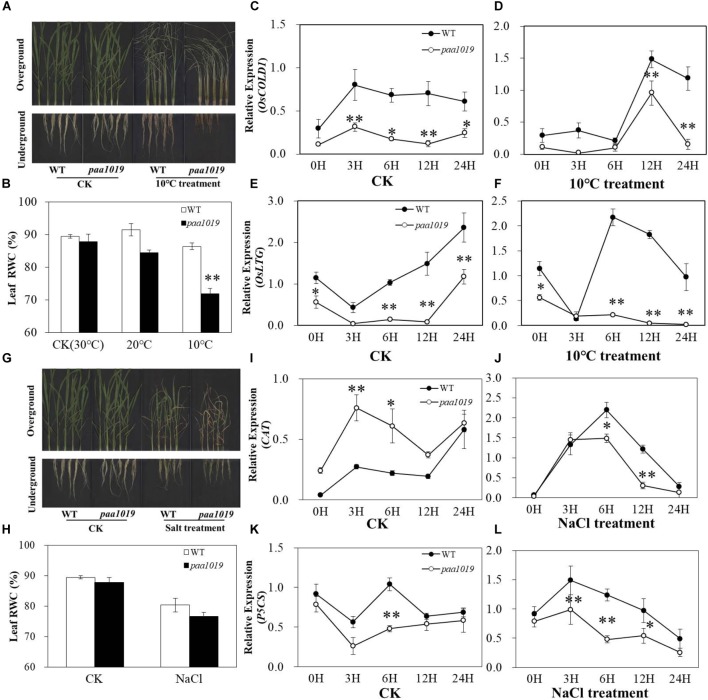
*paa1019* plants are more sensitive to cold and salt stresses. **(A)** Phenotype following cold treatment. **(B)** Relative water concentration in leaves. **(C–F)** Relative expression of *OsCOLD1* and *OsLTG*. **(G)** Phenotype following salt treatment. **(H)** Relative water concentration in leaves. **(I–L)** Relative expression of *CAT* and *P5CS*. ^∗^*p* < 0.05, ^∗∗^*p* < 0.01.

*OsCIPK31*, a member of the *OsCIPK* family, encodes a CBL-interacting protein kinase involved in germination and seedling growth under abiotic stress conditions in rice (Piao et al., 2010). PAA is relatively common and affected by environmental factors during grain production. To further elucidate the multifaceted functions of *OsCIPK31* in rice, we evaluated the performance of *paa1019* under cold and salt stress conditions during the seedling stage. Under normal growth conditions in a hydroponic environment, no significant differences in growth rate or morphological phenotype were detected between WT and *paa1019*. However, after 5 days of cold treatment (10°C), *paa1019* leaves were rolled and droopy, whereas those of WT plants remained upright, although a few rolled leaves were observed. Additionally, *paa1019* roots were more severely wizened than those of WT plants (Figure 5A). To determine the water deficit response quantitatively, the relative water content (RWC) was determined for detached leaves. In response to cold stress, *paa1019* plants lost water faster than WT plants under both 20 and 10°C treatments (Figure 5B). Expression of two cold stress-inducible genes was analyzed. *OsCOLD1* has been reported to play a critical role in cold stress tolerance (Ma Y. et al., 2015). As shown in Figure 5C, *OsCOLD1* expression in WT plants was higher than in *paa1019* under normal conditions, and this remained the case after cold treatment (Figures 5C,D). Consistently, a significant decrease in the expression of *OsLTG1*, another cold-inducible gene, was observed in *paa1019* plants under normal and cold treatments (Figures 5E,F). Abiotic stress usually results in oxidative damage and accumulation of ROS, and the balance between production and removal of ROS is mainly determined by ROS-scavenging enzymes such as catalase (CAT) and glutathione reductase (GR). We therefore examined the activity of these two enzymes. The activity of CAT and GR in *paa1019* was reduced following cold stress (Supplementary Figures S8A,B), suggesting an important role for *OsCIPK31* in the cold stress response. To further assess whether the abortion phenotype in *paa1019* was associated with temperature, *paa1019* plants were grown at different times of the year, and a dramatic negative correlation between abortion rate and average daily temperature was apparent (Supplementary Figure S7).

Consistent with the cold treatment results, we found that the development of *paa1019* plants was arrested more seriously after 5 days of exposure to 100 mM NaCl, and the leaf RWC of WT plants was higher than that of *paa1019* (Figures 5G,H). Lower NaCl dosage led to a shorter lethal period in *paa1019* plants (Supplementary Figure S9). Furthermore, we analyzed the expression levels of two salt stress-inducible genes, *OsP5CS* and *OsCAT*, and compared with WT, expression of *OsP5CS* was consistently downregulated in *paa1019* in both control (CK) and salt treatments (Figures 5K,L). Consistently, a significant decrease in *OsCAT* expression was observed in *paa1019* plants following salt treatment. (Figures 5I,J), indicating a reduction in the salt tolerance of *paa1019* plants. CAT and GR activities in *paa1019* plants were also reduced at all NaCl concentrations except 50 mM, in which GR activity was increased (Supplementary Figures S8C,D). These results demonstrated that *OsCIPK31* positively regulates salt stress tolerance in rice.

### Expression Profiling of *OsCIPK31*

**FIGURE 6:**
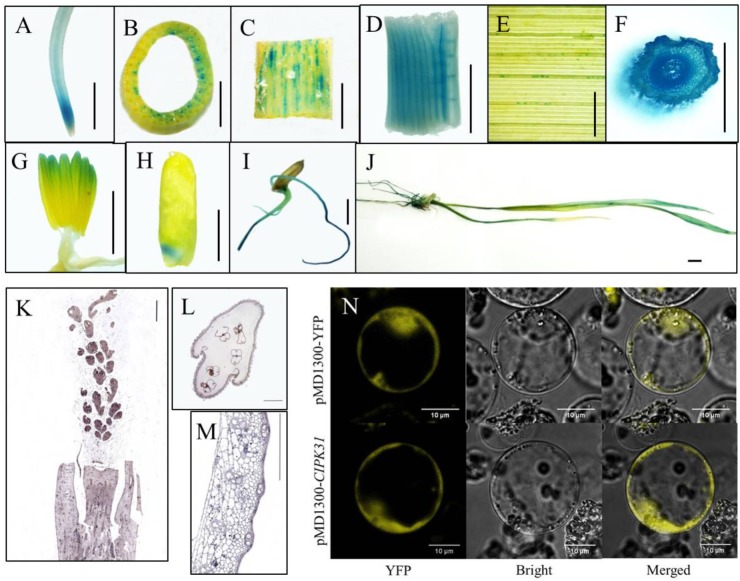
Analysis of *OsCIPK31* expression. **(A–J)** Detection of GUS activity in *OsCIPK31* promoter:GUS transgenic plants. GUS staining was performed using more than three independent transgenic lines with similar patterns. Primary roots **(A)**, stems **(B,C)**, center of young leaves **(D)**, adult leaves **(E)**, young stem nodes **(F)**, stamens **(G)**, embryos of seeds **(H)**, germinating seeds **(I)**, seedlings **(J)**. **(K–M)** *In situ* hybridization of an OsCIPK31-specific probe with a longitudinal section of young panicle **(K)**, and cross-sections of spikelets **(L)** and stems **(M)**. **(N)** Subcellular location of the OsCIPK31 protein. Scale bar = 0.5 cm **(A–J)**, 500 μm **(K–M)** and 10 μm **(N)**.

To explore the spatiotemporal expression of *OsCIPK31* in rice, we examined expression in different tissues by qRT-PCR. The results indicated that *OsCIPK31* was expressed in all examined tissues. The highest expression was found in young roots, and expression was lowest in mature roots. Expression was relatively high in leaves, stems, stamens, and panicle necks, and lowest in developing panicles. However, expression levels were increased during panicle development (Supplementary Figure S10). To further examine the tissue-specific expression pattern of *OsCIPK31*, we generated stable transgenic rice plants expressing the β-glucuronidase (GUS) gene under the control of the native promoter of *OsCIPK31*. GUS histochemical activity was detected in various plant tissues, especially in transport tissues. Strong GUS staining was detected in the meristem zones of primary root and stem (Figures 6A–C), whereas faint staining was observed in veins of adult leaves (Figure 6E). No GUS activity was detected on the surface of young panicles, but obvious staining was observed in the stamens (Figure 6G). Young leaves and young stem nodes showed strong staining (Figures 6D,F). Strong GUS staining was also detected in embryo, radicle, and crown root tissue in germinating seeds (Figures 6H–J).

To further determine the expression of *OsCIPK31*, RNA *in situ* hybridization was performed using developing panicles, stems, and spikelets, and the results demonstrated the preferential expression of *OsCIPK31* in the stamens of spikelets and the hull of vascular tissues in stems (Figures 6K–M). Subcellular location analysis in the rice protoplast indicated that OsCIPK31 was localized to the cytoplasm (Figure 6N).

### Identification of Differentially Expressed Genes (DEGs) Related to PAA Regulation

**FIGURE 7:**
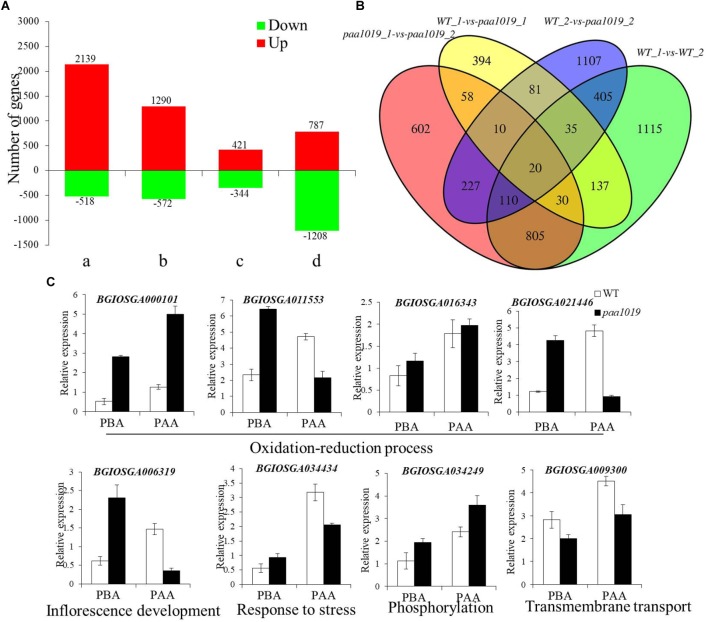
Differentially expressed genes (DEGs) in panicles of WT and *paa1019* plants. **(A)** Number of DEGs in host tissues. Green and red bars indicated down- and upregulated genes, respectively. a, b, c, and d represent WT-2 cm vs. WT-6 cm, paa1019-2 cm vs. paa1019-6 cm, WT-2 cm vs. paa1019-2 cm, and WT-6 cm vs. paa1019-6 cm, respectively. 765 significantly dysregulated genes with a fold change (FC) > 2 and a *p*-value less than 0.05 was identified. Among these, 421 were upregulated and 344 were downregulated in mutant panicles before degradation. By contrast, 1995 significantly dysregulated genes, including 787 upregulated and 1208 downregulated genes, were identified between WT and mutant panicles after degradation. A total of 2657 genes, including 2139 upregulated and 518 downregulated genes, were observed in WT groups, and 1862 genes, including 1290 upregulated and 572 downregulated genes, were identified in mutant groups. **(B)** Venn diagram showing genes common to WT-1, WT-2, paa1019-1, and paa1019-2 groups (represented by WT-2 cm, WT-6 cm, paa1019-2 cm, and paa1019-6 cm, respectively). **(C)** Analysis of the expression of common genes.

To elucidate the possible regulatory mechanisms of *OsCIPK31* in panicle development, we performed transcriptome profiling of WT and *paa1019* panicles at 2 and 6 cm length using RNA-seq. Young panicle tissue was collected at two stages encompassing the entire PAA process. Principal component analysis (PCA) and correlation analysis indicated that the RNA sequencing data were of high quality, with sufficient sequencing depth for subsequent analysis of differential expression (Supplementary Figures S11A–D). Differential expression and analysis of differences in each gene between WT and *paa1019* plants, during panicles before abortion (PBA) and panicles following abortion (PFA), showed different groups with unequal dysregulation of genes with a fold change (FC) > 2 and a *p*-value less than 0.05 (Figure 7A).

To identify DEGs common in both WT and *paa1019* panicles, we compared DEGs in each group. The Venn diagram revealed 20 specific genes with diverse regulatory patterns, details of which are shown in Supplementary Table S3 and Figure 7B. Functional analysis showed that four genes (*BGIOSGA000101*, *BGIOSGA011553*, *BGIOSGA016343*, and *BGIOSGA021446*) were predicted to be essential in the oxidation-reduction and hydrogen peroxide catabolic processes, and response to oxidative stress in plants. Based on GO term analysis, *BGIOSGA006319* could play important roles in inflorescence development. Meanwhile, *BGIOSGA034434* and *BGIOSGA009926* are known to respond to various stresses. The rest of the dysregulated genes were divided into three groups, all of which were related to lipids. *BGIOSGA009099*, *BGIOSGA021541*, *BGIOSGA023124*, and *BGIOSGA029706* are predicted to be related to cell wall and membrane biogenesis. *BGIOSGA004346*, *BGIOSGA009300*, and *BGIOSGA031591* are regulators of transmembrane transport. *BGIOSGA034249* is involved in phosphorylation-related signal transduction. Further qRT-PCR analysis verified that the expression of selected genes was found different between WT and *paa1019* panicles (Figure 7C), and these differences are consistent with the RNA-seq analysis. The results implied that *OsCIPK31* regulation is related to the inflorescence development, responses to various stresses, and ROS accumulation by controlling the expression of specific genes.

### Functional Enrichment in Response to *OsCIPK31* Mutation

**FIGURE 8:**
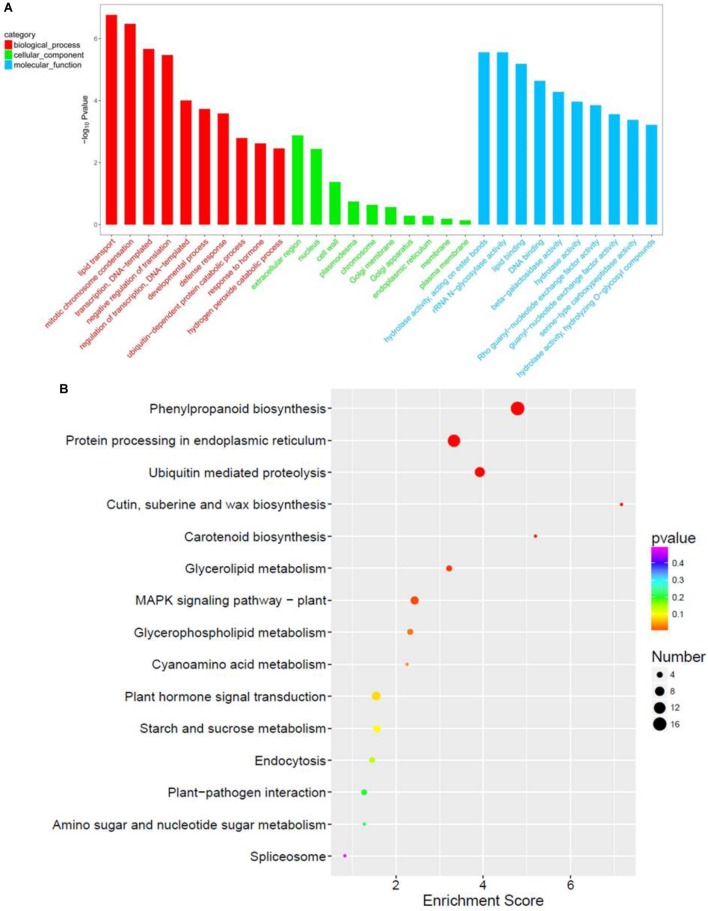
Functional enrichment in response to *OsCIPK31* mutation. **(A)** Comparison of GO enrichment analysis. The top 10 significantly enriched GO terms for biological process (BP, cellular component (CC) and molecular function (MF) in PAA relative to normal panicle development in WT plants are shown in red, green, and blue, respectively. **(B)** Comparison of Kyoto Encyclopedia of Genes and Genomes (KEGG) pathway enrichment. The top 20 significantly enriched KEGG pathways in PAA relative to normal panicle development in WT plants are shown. The *x*-axis represents the enrichment factor for each pathway, and the *y*-axis shows the KEGG pathway names. The size of the circle represents the number of genes involved in each significant pathway. The color represents the significance level of the -log (*p*-value), from red (most significant) to green (least significant).

GO term analysis revealed that biological process (BP), cellular component (CC), and molecular function (MF) were more enriched in *paa1019* panicle abortion than in WT panicle development (Figure 8A). For the top 10 BP terms, the most relevant was lipid transport, consistent with Venn diagram analysis, and basic genetic processes also scored highly. Association of BP terms with defense and hormonal response revealed, abiotic stress and IAA are involved in the regulation of PAA phenotype in *paa1019* mutant. Interestingly, the top 10 CC terms were associated with integral membrane components, whereas MF terms were enriched in lipid/protein responses to stress (Figure 8A). Also, based on Kyoto Encyclopedia of Genes and Genomes (KEGG) pathway analysis (Figure 8B), DEGs were mainly associated with lipid/protein regulation pathways such as phenylpropanoid biosynthesis (osa00940), ubiquitin-mediated proteolysis (osa04120), MAPK signaling pathways in plants (osa04016), and plant hormone signal transduction (osa04075; Figure 8B).

**FIGURE 9:**
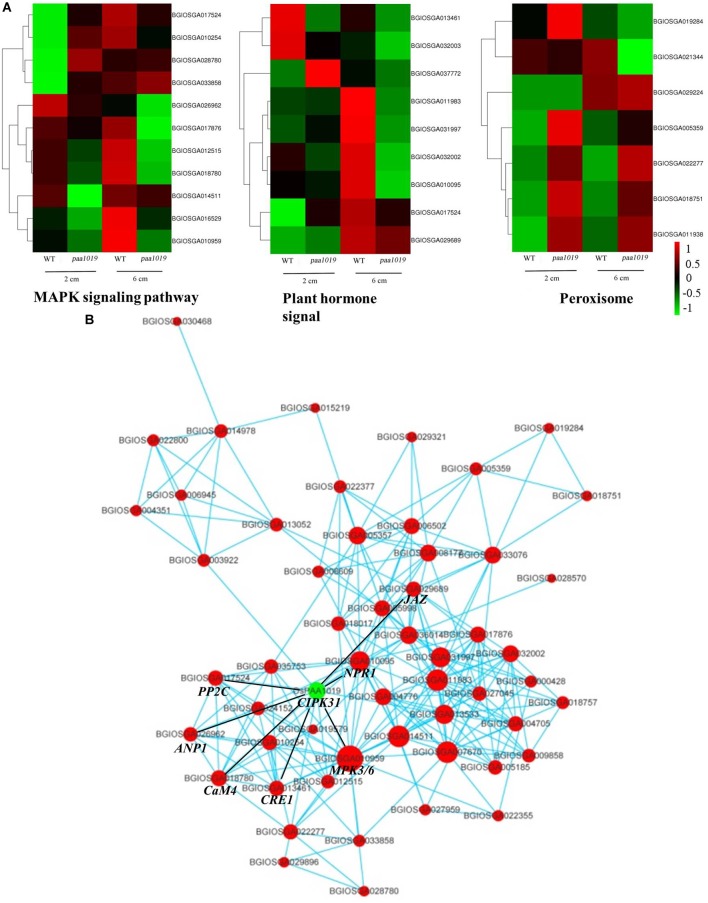
Interaction networks involving *OsCIPK31* and the most significantly enriched pathways. **(A)** Comparison of DEGs in different pathways between WT and *paa1019* plants. **(B)** Protein-protein interaction (PPI) networks involving *OsCIPK31* and the most significantly enriched pathways (the MAPK signaling pathway, plant hormone signal transduction, peroxisome, and alpha-linolenic acid metabolism).

Furthermore, several representative modules for lipid/protein responses to stress were observed in the cluster analysis, and relevant genes were differentially expressed in different samples (Figure 9A). Furthermore, qRT-PCR showed different expression of important genes in *paa1019* than WT panicles, which are regulating different phytohormones involved in the transduction of MAPK signaling pathway (Supplementary Figure S12). These results strongly suggest the MAPK signaling pathway and phytohormone signal transduction are regulated by *OsCIPK31*. To further explore the connection between these genes and pathways, gene regulatory networks based on protein-protein interactions (PPIs) were constructed. The relative size of each node presents the strength of the correlation. The networks revealed that most genes potentially interacting with *OsCIPK31* belong to the MAPK signaling pathway (*MPK3/6*, *CaM4*, *PP2C*, and *ANP1*) and the plant hormone signal transduction pathway (*NPR1*, *CRE1* and *JAZ*; Figure 9B). These results suggest that *OsCIPK31* might be involved in the regulation of expression of those genes that are involved in MAPK signaling and phytohormonal transduction pathway.

## Discussion

Panicle development is crucial for grain yield in rice, and PAA often causes yield losses during rice production. Commencement of PAA is complex and probably associated with environmental conditions. In this study, we identified *OsCIPK31*, which encodes CBL-interacting protein kinase OsCIPK31. *OsCIPK31* is expressed mainly in roots in response to environmental signals, and regulates the development of panicle apical spikelets, and it can also be influenced with content of phytohormones in the panicles.

Calcineurin B-like proteins (CBLs) are Ca^2+^ sensors in plant cells that regulate the activity of CBL-interacting protein kinases (CIPKs) (Luan et al., 2002; Kolukisaoglu et al., 2004). CIPKs transmit the calcium-mediated signal downstream, generally by phosphorylating target proteins such as transcription factors or transporters/channels, ultimately triggering appropriate responses. In *Arabidopsis*, 10 CBLs and 26 CIPKs have been identified (Weinl and Kudla, 2009; Hashimoto et al., 2012), whereas 10 CBLs and 30 CIPKs have been found in *O. sativa* (Albrecht et al., 2001; Kolukisaoglu et al., 2004). Since the discovery of CBL-CIPKs, most of them have been associated with abiotic stress signaling (Cuéllar et al., 2010; Hu et al., 2012; Ji et al., 2013; Kanwar et al., 2014), and some are also believed to be involved in biotic and/or oxidative stress and in developmental pathways as well (Xiang et al., 2007; Piao et al., 2010; Zhang et al., 2010, 2013). In rice, OsCIPKs are involved in abiotic stress signaling, and 20 OsCIPKs respond to at least one type of any abiotic stress, such as drought, salt, low temperature, or abscisic acid (ABA) (Xiang et al., 2007). *OsCK1* (*OsCIPK31*) is influenced by diverse signaling of cold, salt, light, cytokinins, sugars, and calcium upregulates transcript levels of *OsCIPK31*. The *OsCIPK31* protein phosphorylates itself to a generic substrate, myelin basic protein, in the presence of Mn^2+^ (Kim et al., 2003). Overexpression of *OsCIPK31* (*OsCIPK3*) enhances cold tolerance (Xiang et al., 2007), as *OsCIPK31* modulates responses to abiotic stress during seed germination at various seedling stages, and regulates the expression of stress-responsive genes (Piao et al., 2010). However, further detailed analysis of the role of *OsCIPK31* in abiotic stress in rice is needed, with particular attention on crosstalk and the interconnection of CIPK signaling with other signaling pathways such as mitogen-activated protein kinases (MAPKs), phytohormones such as ABA, jasmonic acid (JA), and salicylic acid (SA), and auxin-mediated signaling networks. However, it is not known whether *OsCIPK31* is involved in developmental pathways especially in panicle development of rice.

### PAA Exhibits Apical Dominance and Is Regulated by Phytohormones, Especially Auxin

Panicle architecture is regulated at several positions where primary branches are formed by genes such as *lax1* (Komatsu et al., 2003; Oikawa and Kyozuka, 2009), *moc1* (Li et al., 2003), and *SP1* is an essential regulator that controls panicle branch elongation processes other than the initiation of branch primordia (Li et al., 2009). However, we found that regulation of PAA development was spatiotemporal. Unlike WT plants in which all young branches elongate normally, development of the top spikelets in the *paa1019* mutant (which has young panicles of ∼4 cm) was clearly arrested. *OsCIPK31* appears to be an essential regulator that controls panicle branch elongation, especially spikelet development other than the initiation of branch and spikelet primordia. Furthermore, the degree of abortion was correlated with spikelet location.

In general, rice florets on branches at the top of young panicles are the first to appear, and for each branch, apical spikelets are predominant. This implies “apical dominance” during panicle differentiation, and the same trend was also observed in flag leaves (Supplementary Figure S2A). It is widely believed that auxin accumulation in the apical meristem promotes terminal bud growth and inhibits lateral bud growth. Auxin biosynthesis in the apical bud is a characteristic of polar transport, which occurs in panicles from the top to down. Interestingly, this mode of development is consistent with the spatiotemporal features of PAA observed in the present study. Similarly, polar transport of IAA was found to be reduced in the *cipk6* mutant, which led to hypocotyl expansion and delayed lateral root development (Tripathi et al., 2009). These observations indicate a relationship between the degree of PAA and auxin accumulation. Herein, we found that IAA levels in young panicles of *paa1019* plants were higher than those in WT plants, and BP terms in GO analysis were enriched in responses to plant hormone signals. Also, KEGG pathway analysis showed that many of the identified DEGs were related to phytohormone signal transduction (osa04075). PPI analysis also (Figure 9B) supported that *OsCIPK31* might interact with other factors that are associated with the phytohormonal signal transduction pathway. In addition, vessels of vascular bundles in secondary branches were found to be blocked to varying degrees in *paa1019*, which could explain why material transportation deficiency plummeted. Together, these results suggest auxins affect polar transport, and make a necessary contribution to regulating PAA. In conclusion, auxins appear to affect polar transport and spatiotemporal development of PAA in *paa1019* and hormone signaling could be a major factor in determining the development and distribution of PAA.

### Mutation in *OsCIPK31* Increases Sensitivity to Stress, Accompanying ROS Accumulation in the Panicle

Among the various stresses affecting plant growth and development, abiotic stresses represent a major problem for agricultural production worldwide. PAA is clearly influenced by the external environment. The role of CBL-CIPK signaling in regulating diverse aspects of abiotic stress responses has been thoroughly investigated in *Arabidopsis*. Recently, studies have shown that CIPKs could regulate the plant development by interacting with CBLs or only itself, so CBLs are not indispensable for *CIPK31* functioning (Bender et al., 2018). The CIPK family member CIPK31 is believed to play a role in the regulation of seedling growth in rice. Herein, we found that *OsCIPK31* positively regulates cold and salt stress responses, whereas the degree of panicle abortion was negatively regulated by temperature in rice. These results strongly suggest that mutation in *OsCIPK31* led to increased sensitivity to cold and salt stresses; hence, the development of *paa1019* plants was more susceptible to adverse environmental conditions.

Excessive sensitivity to stress is not usually favorable for plant growth and development, since this can result in higher ROS levels. Indeed, our ROS experiments also indicated excessive H_2_O_2_ accumulation in plants suffered PAA. The H_2_O_2_ signal was stronger in different developmental stages in apical spikelets of *paa1019* plants, and differential expression of genes related to oxidation-reduction and hydrogen peroxide catabolic processes was evident (Figures 7C, 8A).

Usually PMs and cell wall provide a barrier to environmental factors, and are means of plant defense activation by which responses can be initiated (Mongrand et al., 2010). The PM is a medium for exchange of metabolites such as signaling molecules, ions, and hormones (Yadeta et al., 2013). Herein, we found that the PM system of panicle cells was damaged in plants suffering from PAA. The cell wall and cytoplasmic membrane of glumes and stamens in *paa1019* were deformed during booting and heading stages. Transcription analysis further confirmed that lipid/protein pathways were affected by *OsCIPK31*. H_2_O_2_ accumulation appears to lead the damage of cellular structures, interrupting the growth of young spikelets, and eventually resulting in cell death.

Mutation in *OsCIPK31* increases sensitivity to stresses and disrupt the balance of metabolic pathways of some phytohormones. *Paa1019* is susceptible to some environmental stresses that is why the PAA showed a degree of phenotypes under different environment.

### Synergistic Effect Between *OsCIPK31* and MAPK Pathway Signaling in Response to Environmental Stress

Mitogen-activated protein kinase cascades are involved in several aspects of plant physiology, including cell division, plant growth and development, plant resistance to pathogens, and plant response to abiotic stresses (Liu and He, 2017). There is a complex relationship between the MAPK pathway and ROS. On one hand, ROS activates and regulates MAPK cascades; while on the other hand MAPK cascades could also regulate ROS-related genes and result in initiation of ROS responses. Collectively, ROS and MAPK cascades could induce cell death collectively (Liu and He, 2017). CIPKs have been found to interact with and target a large number of partners including phosphatases (Ohta et al., 2003), transporters/channels (Sánchez-Barrena et al., 2012), transcription factors (Song et al., 2005), and enzymes (Drerup et al., 2013). Here, we also found that PAA was accompanied by programmed cell death (PCD) in *paa1019*. Excessive ROS accumulation was observed in the young panicle of *paa1019* mutant and it also exhibited hypersensitivity to abiotic stress. Furthermore, RNA-seq analysis of WT and *paa1019* revealed numerous PAA-related MAPK signaling pathway DEGs. PPI analysis (Figure 9B) predicted that *OsCIPK31* might interact with factors that are associated with the MAPK signaling pathway. Based on the above findings, we predicted that *OsCIPK31* and MAPK pathway have a synergistic effect to lead to ROS accumulation in response to stresses.

**FIGURE 10:**
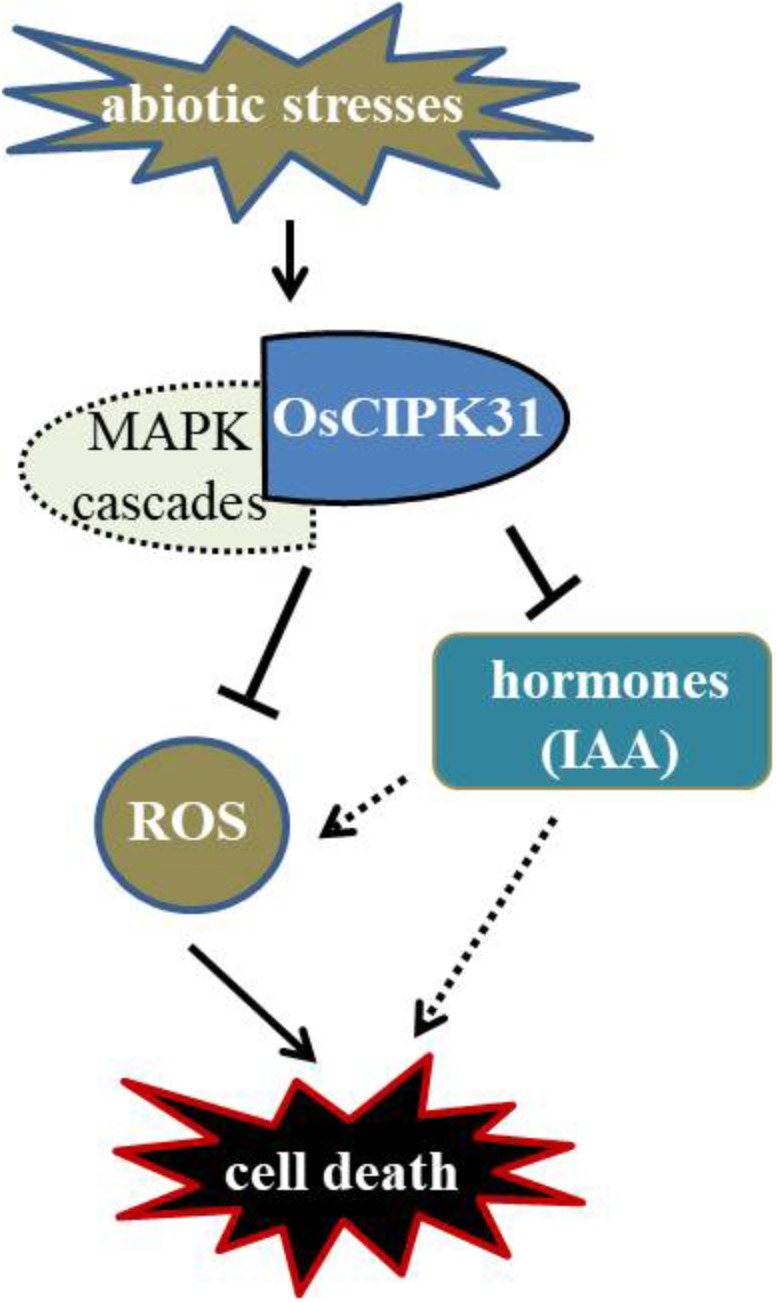
Putative regulation of panicle development by *OsCIPK31*. *OsCIPK31* perceives the response against stresses and regulates ROS accumulation, which might be collaboration with MAPK cascades. Meanwhile *OsCIPK31* regulates IAA phytohormone distribution in the panicle, and excessive IAA might lead to the ROS accumulation in the apical spikelet, and ROS regulates PCD of the panicle.

Previous studies have greatly appreciated the role of CIPKs in regulation of various biological processes such as abiotic stress, plant development, and ROS signaling (Pandey, 2008; Batistiè et al., 2012; Tang et al., 2012). *OsCIPK31* plays an important role in the regulation of seedling growth and response to stresses in rice (Piao et al., 2010). In this study, we demonstrated that *OsCIPK31* is a key factor required to maintain sustained growth of the panicle, and the mutation in *OsCIPK31* leads to PAA phenotype. As abortion of spikelets is particularly seen under unfavorable environmental conditions (Itoh et al., 2005; Kato et al., 2008), we found dysregulation is caused by the disruption of the *OsCIPK31* function due to excessive accumulation of ROS, which ultimately leads to cell death in rice panicle. MAPK cascades also might take part in response to environmental stresses and regulate ROS metabolism. There is a relationship between PAA distribution and IAA phytohormone accumulation in the panicle (Figure 10). Thus, our discovery provides novel insights into panicle development by *OsCIPK31* likely through responding to stresses in rice and other species.

## Materials and Methods

### Plant Materials and Growth Conditions

The stably inherited mutation in *paa1019* was derived from the progeny of the excellent indica maintainer line Yixiang 1B, seeds of which were treated with 1% EMS. Yixiang 1B plants are represented as WT plants throughout this work. The 02428 japonica cultivar was obtained from Jiangsu Province Academy of Agricultural Sciences, and *paa1019* and F_1_ plants were self-crossed to generate the F_2_ population for gene mapping of PAA1019. The *paa1019* mutant was also backcrossed with WT Yixiang 1B and self-crossed to create the BC_1_F_2_ population. Rice plants were cultivated under natural conditions in an experimental field at Sichuan Agricultural University Rice Research Institute, or at Lingshui, Hainan Province, China.

### Determination of Antioxidase Activities

A 0.3 g fresh sample was homogenized in 5 mL of 50 mM TRIS-HCl buffer (pH 7.0) containing 1 mM EDTA, dithiothreitol (DTT), acrylonitrile styrene acrylate copolymer (AsA), reduced glutathione (GSH), and 5 mM MgCl_2_. Catalase (CAT) activity was determined by measuring the consumption of H_2_O_2_ at 240 nm according to the method of Aebi with slight modifications. The reaction mixture consisted of 2.9 mL 50 mM TRIS-HCl buffer (0.1 mM EDTA, pH 7.0), 50 μL 750 mM H_2_O_2_, and 50 μL enzyme extract. The consumption of H_2_O_2_ was monitored by the decrease in absorbance of light at 240 nm over 3 min. Activity is expressed in units (mM of H_2_O_2_ decomposed per min) per g of formula weight (FW).

Glutathione reductase (GR) activity was assessed as described by Foyer and Halliwell (Foyer and Halliwell, 1976) with minor modifications. The 3.0 mL assay mixture consisted of 450 μL of enzyme extract, 2.34 mL 50 mM TRIS-HCl buffer (0.1 mM EDTA, 5 mM MgCl_2_, pH 7.5), 60 μL 10 mM NADPH, and 150 μL 10 mM oxidized glutathione. The NADPH oxidation rate was monitored by measuring the decrease in absorbance at 340 nm. GR activity is expressed as the amount of enzyme needed to oxidize 1 μmol of NADPH per min per g of FW.

### Stress Treatments

Seeds were surface-sterilized with 2% H_2_O_2_ for 24 h and washed with flowing water for 1 day. After sterilization, seeds were germinated in nutrient solution at 26°C for 16 h under light conditions, then at 22°C for 8 h in the dark. Two-week-old seedlings were used for stress treatments and subjected to NaCl (50, 100, or 150 mM) and temperatures of 10 or 20°C.

### Genetic Analysis and Map-Based Cloning

To identify genes involved in normal panicle and spikelet development in rice, a mutant library of 10,431 accessions of indica cultivar Yixiang 1B was generated using ethyl methane sulfonate (EMS) mutagenesis and screened for PAA mutants. One mutant displaying apical abortion was selected for further genetic and complementation analyses. As detailed below, this mutant was named PAA *paa1019*. When pollinated with WT pollen grains, all *paa1019* F1 progeny displayed the WT phenotype and grew normal panicles. The F_2_ population derived from the cross of 02428 × paa1019 was used for rough mapping using bulk segregation analysis (BSA). A physical linkage map was then constructed using molecular markers near the *PAA1019* locus. The SSR sequences and InDel markers are listed in Supplementary Table S1.

For whole-genome resequencing (Mutmap), the *paa1019* was backcrossed with the WT and self-crossed to generate BC_1_F_2_ population. The equal total DNA of 25 BC_1_F_2_ plants displaying the PAA phenotype was mixed and resequenced by Illumina HiSeq 2500 platform and the WT genome DNA was also sequenced as control. Then raw data (raw reads) of fastq format were first processed through a series of quality control (QC) procedures in-house C scripts. Burrows–Wheeler Aligner (Li and Durbin, 2009) was used to align the clean reads of each sample against the reference genome (93-11 cultivar). ANNOVAR was used to annotate SNP or InDel based on the GFF3 files for the reference genome (Wang et al., 2010). Finally, the difference of the SNP index of two pools was calculated as the delta SNP index.

### DNA Extraction, Primer Design, and PCR/qRT-PCR

Total DNA from parental and mutant lines was extracted using the cetyltrimethylammonium bromide (CTAB) method. The SSR markers were designed according to information in the Gramene database^1^. The InDel primers were designed based on the alignment of the reference genome of 93–11 with sequence of the indica rice cultivar^2^ and Nipponbare japonica rice cultivar^2^. The PCR mixtures (20 μL) contained the following components: 2 μL DNA (50 ng/μL), 2 μL primers (10 μmol/μL), 0.3 μL dNTPs (10 mM), 0.2 μL Taq DNA Polymerase (5 U/μL), and 13.5 μL ddH_2_O. Amplifications were performed using the following procedure: 5 min at 95°C, followed by 33 cycles of 30 s at 95°C, 30 s at 55°C, and 1 min at 72°C, with a final extension at 72°C for 10 min. The PCR products were separated on 3% agarose gels and visualized by ethidium bromide (EB) staining.

Total RNA was extracted from different tissues using TRIZOL (Invitrogen, Carlsbad, CA, United States). First-strand of cDNAs was synthesized using a PrimeScript RT Reagent Kit with gDNA Eraser (TaKaRa Bio Inc., China). Then, qRT-PCR was performed using a C1000 thermal cycler and a Bio-Rad CFX96 Real-Time System (Bio-Rad, Hercules, CA, United States). Relative expression levels of genes were measured relative to *OsActin1* and calculated using the 2^-ΔΔCt^ method. The values are presented as means ± SD of three biological repeats. The primers are listed in Supplementary Table S2.

### mRNA Sequencing and Analysis of RNA-Seq Data

Analysis of RNA-seq data was performed using the following standard protocol (Trapnell et al., 2012). Total RNA was extracted from 2 cm panicles from control samples, 6 cm panicles from WT plants (WT-1 and WT-2), and from 2 and 6 cm panicles of experimental samples (mutants *paa1019-1* and *paa1019-2*) using TRIZOL. A total of 12 sequencing samples were treated, and each group included three biological replicates. RNA sequencing was performed by OE Biotechnology (Shanghai, China), and sequencing data were retrieved through the standard Illumina pipeline with custom and default parameters. All the 12 raw reads were deposited into the NCBI Sequence Read Archive (SRA) database with accession number SRX4908949∼4908960. Additional filtration was performed by trimming adapter and low-quality sequences. For each sample, STAR software was used for sequence alignment of preprocessed sequences and reference genome sequences (OE Bio Inc., Shanghai, China). Sequences of mouse genes were downloaded from the Ensemble database^2^. HTSeq software was used to analyze the original sequences of known genes for all the samples (OE Bio Inc., Shanghai, China), and expression of known genes was calculated using the fragments per kilobase of transcript per million fragments mapped (FPKM). Clusters were analyzed by principal component analysis (PCA), and differentially expressed genes (DEGs) were analyzed by DESeq, with cut-offs *p*-value ≤ 0.05 and fold change (FC) ≥ 2. The distance metric of the Pearson correlation, average linkage, and hierarchical clustering were calculated for the DEGs, and functional annotation was performed using gene set enrichment analysis (GSEA), which includes Gene Ontology (GO) and Kyoto Encyclopedia of Genes and Genomes (KEGG) pathway enrichment. Cytoscape was used to reconstruct the regulatory networks^3^.

### Scanning Electron Microscopy

Shoot apex tissue from the vegetative apices of WT and *paa1019* plants was collected and dissected carefully under a sterol microscope, and subsequently fixed in 2.5% glutaraldehyde in 0.1 M phosphate-buffered saline (PBS; 4 mM sodium phosphate, 200 mM NaCl, pH 7.2) at 4°C overnight. After rinsing twice with PBS, the samples were rinsed in 4% (w/v) sucrose solution for 5 min, then dehydrated using a graded series of ethanol (30, 50, 70, 80, 90, 95, and 100%). Finally, the samples were dried in a critical-point drier, sputter-coated with platinum, and observed under bright-field by scanning electron microscopy (Inspect, FEI, United States).

### Histological Analysis and RNA *in situ* Hybridization

Young panicles from WT and *paa1019* plants at different developmental stages were collected for ROS analysis using 3,3′-diaminobenzidine (DAB) staining to measure H_2_O_2_ accumulation as described previously (Zhu et al., 2016). Trypan blue staining assays were also performed as described previously with minor modifications (Zhu et al., 2016). Briefly, the samples were incubated in lactic acid-phenol-trypan blue solution overnight and destained with buffer (ethanol:acetic acid, 84:16, v/v) for 24 - 48 h. Evans blue staining was performed according to the manufacturer’s instructions^4^. Paraffin sectioning was done according to previous reports (Ma et al., 2017).

For RNA *in situ* hybridization, a gene-specific region of *OsCIPK31* mRNA was selected as the digoxigenin (DIG)-labeled RNA probe (Roche), and labeled with DIG at the 5′ end. RNA *in situ* hybridization with detectable probes was performed as described previously (Liu et al., 2015). The probes are listed in Supplementary Table S4.

### GUS Staining Assay

β-glucuronidase (GUS) activity was detected according to the instructions supplied with the GUS staining kit^5^. First, X-gluc powder was dissolved in X-gluc solution (1 mL) and stored at -20°C. Working solution was then obtained by mixing X-gluc with GUS buffer (1:50). The plant tissues were collected and fixed in 90% acetone for 20 min, then vacuum-infiltrated in working solution and incubated at 37°C overnight. After the incubation, the tissues were cleaned in destaining buffer (ethanol:acetic acid, 84:16, v/v) at room temperature. Finally, clean tissue samples were observed under a dissecting microscope (Leica S8APO, Germany).

### Subcellular Localization of OsCIPK31

To investigate the subcellular localization of OsCIPK31, the coding sequence of OsCIPK31 was amplified with the primer pair pMD1300-CIPK31-YFP-1 and pMD1300-CIPK31-YFP-2 from the wild-type plant and then cloned into the pMD1300 vector to generate a N-terminal fusion with the yellow fluorescent protein (YFP) under control of the CaMV 35S promoter, resulting in the pMD1300-CIPK31-YFP construct. Rice protoplasts were isolated from the indica cv. 9311 cultivar seedlings leaves (7–15 days after germination with dark treatment, 30°C), and were transformed with the plasmids pMD1300-CIPK31-YFP following the method as described previously (Ren et al., 2013). YFP fluorescence was detected using a confocal laser scanning microscope (Olympus FluoView FV1000, Japan).

### Material Content Determination

To measure chlorophyll content, flag leaves (0.2 g) were collected from WT and *paa1019* plants and soaked in 20 mL of 95% alcohol for 48 h in the dark, then examined spectrophotometrically at 665 and 649 nm (in triplicate). The following formulae were then applied: Chl a absorbance = (13.95 × OD_665_) – (6.88 × OD_649_); Chl b absorbance = (24.96 × OD_649_) – (7.32 × OD_665_); Chlorophyll content (mg/g) = (C × V × D)/(1000 × W) (where C = absorbance, V = volume, D = dilution, and W = weight of sample).

The water content (WC) of seedlings was calculated using the following formula: WC [%] = [(fresh weight – dry weight)/fresh weight] × 100 (Kim et al., 2005). Determination of IAA content was carried out using the enzyme-linked immunosorbent assay (ELISA) method as previously described (Teng et al., 2006).

### Vector Construction and Plant Transformation

A 5.7 kb genomic fragment containing the 2 kb native promoter and the entire *CIPK31* gene sequence was cloned into the pBWA(V)BII vector to generate the pBWA(V)BII-PROCIPK31:CIPK31 complementation construct. To generate the CIPK31-CRISPER/Cas9 construct, two sites of the 20 nt guide RNA (gRNA) targeting the CIPK31 coding sequence were amplified and cloned into the pCRISPR/CAS9–MT vector as described previously (Ma X. et al., 2015). For promoter-GUS assays, the genomic fragment of the complementation construct was amplified and cloned into the pBWA(V)BII-GUS vector. The promoter and full-length coding sequence of Os*CIPK31* were separately amplified with pMD1300-CIPK31-YFP-1 and pMD1300-CIPK31-YFP-2 markers, and the resulting PCR products were cloned into the pMD1300-YFP vector. All the resulting constructs were introduced into rice using the *Agrobacterium tumefaciens*-mediated transformation method. Primers used for vector construction are listed in Supplementary Table S5.

## Author Contributions

XW designed and performed the project. YP, FH, and QB performed the analysis of agronomic traits, histochemical staining, stress treatment, and assessed rice stress resistance. YP, FH, and AA carried out genetic analysis, map-based cloning assay. YP and TW performed the mutation analysis and sequence analysis. YP, YL, and PX performed the vector construction and plant transformation. XD, CG, HZ, YP, and XC assisted in the data analysis. YP, AA, and XW wrote the manuscript. All the authors read and approved the final manuscript.

## Conflict of Interest Statement

The authors declare that the research was conducted in the absence of any commercial or financial relationships that could be construed as a potential conflict of interest.

## Footnotes

1 http://www.gramene.org/microsat

2 http://plants.ensembl.org/Oryza_indica/Info/Index

3 www.cytoscape.org/

4 www.solarbio.com

5 www.biosharp.cn

## Supplementary Material

Figure S1 Phenotypes of the *paa1019* mutant. **(A-D)** Agronomic traits of wild-type (WT) and *paa1019* mutant plants. **(A)** Duration. **(B)** Tilling number. **(C)** Secondary branch number. **(D)** Abortion rate. Statistical analysis was performed using Student’s *t-*tests, ^∗^*p* < 0.05, ^∗∗^*p* < 0.01.

Figure S2 Phenotype of leaves. **(A)** The color of leaves in paa1019 is more yellow than in WT plants. **(B-E)** Content of chlorophyll **(B)**, carotene **(C)**, net photosynthetic rate **(D)**, and organic acids **(E)**. ^∗^*p* < 0.05, ^∗∗^*p* < 0.01.

Figure S3 The alternative splicing of *PAA1019* gene. **(A)** The pair of primers used for RT-PCR analysis. The forward and reverse primers were located in 1st and 5th exons, respectively. **(B)** RT-PCR analysis showing the presence of the abnormal size transcript of *paa1019*. The primers were used for amplification that is given in **(A)**, where larger transcript was of WT (due to 2nd exon) and smaller transcript was of *paa1019*. The genomic DNA was used as a control. **(C)** The alternative splicing of *PAA1019* gene in *paa1019*. The boxes are indicating exons, whereas the dotted lines indicate base sequences. The deleted region is highlighted in red (2nd exon), whereas base substitution is highlighted in green (that was located between the boundary of 1st intron and 2nd exon). **(D)** Sequencing and alignment of CDS obtained in **B** (WT and *paa1019*) showed the deletion of entire 2nd exon in *paa1019.* **(E)** Sequencing and alignment of DNA obtained in **(B)** revealed a base substitution from G to A in *paa1019*.

Figure S4 Relative expression of *OsCIPK31* in WT, *paa1019*, and complementation lines. *CE1-CE3* represent the three complementation transgenic lines. Statistical analysis was performed using Student’s *t*-tests.

Figure S5 The sgRNA target sequence analysis of the knock-out lines. Different mutation at the target site in three representative knock-out lines generated by the CRISPR/Cas9 technology. The filled bars and lines are indicating exons and introns of *OsCIPK31*, respectively. The knock-out target region was located in the fourth exon of *OsCIPK31*. Mutation types of *KO1* and *KO3* (knock-out lines) were substitution. While the mutation type of *KO2* was insertion. These mutations led to the premature termination of translation in three lines (the terminator codon is not shown in *KO2* sequence). The black line indicates sgRNA target sequences. The red and green triangles are indicating the substitutive and inserted sites, respectively. *OsCIPK31_DNA*, *OsCIPK31_CDS*, and *KO1/2/3* are indicating *OsCIPK31* genomic, CDS, and three knock-out transgenic lines cDNA sequences, respectively. *KO1/2/3 coding amino acid* is indicating the corresponding amino acid sequences, where red color is depicting changed amino acid and ^∗^ to a termination codon.

Figure S6 Development of flowers is arrested in the degraded spikelets of *paa1019* plants. **(A–J)** Phenotype of flower development observed at different growth stages. **(A,B)** = 20 DBF; **(C,D)** = 15 DBF; **(E,F)** = 10 DBF; **(G,H)** = 5 DBF; **(I,J)** = flowering stage; **(K-L)** KI staining of pollen. DBF indicated days before flowering. Scale bar = 0.2 cm **(A-J)**.

Figure S7 The abortion rate is influenced by the environmental temperature.

Figure S8 Activity of CAT and GR enzymes following different treatments. **(A,B)** Activity of CAT and GR enzymes following the cold treatments; **(C,D)** activity of CAT and GR enzymes following the salt treatments. ^∗^*p* < 0.05, ^∗∗^*p* < 0.01.

Figure S9 The lethal duration following different treatments.

Figure S10 Relative expression of *OsCIPK31* in wild-type. Tissues including young roots, young leaves, 0–20 cm panicles (40–70 days after germination) and stems, panicle neck, adult roots, adult leaves, stamens (80 days after germination). Rice ACTIN was used as an internal control. Data are presented as mean ± SE (*n* = 3).

Figure S11 Differentially expressed genes (DEGs) between WT and *paa1019* plants. **(A,B)** Heatmap showing the expression patterns of significantly dysregulated genes for WT-2 cm vs. paa1019-2 cm **(A)** and WT-6 cm vs. paa1019-6 cm **(B)** groups. According to DEGs analysis, significantly dysregulated genes displayed a fold change (FC) ≥ 2 and a *p*-value ≤ 0.05. **(C,D)** Volcano plot of DEGs in paa1019-2 cm **(C)** and paa1019-6 cm **(D)** relative to controls. DEGs were selected based on a false discovery rate (FDR) < 0.05 and a |log_2_ fold change| > 1.0. The *x*-axis shows the fold change in gene expression between paa1019-2 cm **(C)** and paa1019-6 cm **(D)** relative to controls, and the *y*-axis shows the –log_10_ (FDR). Blue splashes indicate genes not exhibiting significant differences in expression. Red splashes indicate significantly upregulated genes. Green splashes indicate significantly downregulated genes. Gray splashes indicate unfiltered genes.

Figure S12 Relative expression of genes related to MAPK signaling pathway and phytohormone signal transduction. **(A)** Relative expression of genes related to MAPK signaling pathway. **(B)** Relative expression of genes related to phytohormone signal transduction. Rice ACTIN was used as an internal control. Data are presented as mean ± SE (*n* = 3). ^∗^*p* < 0.05, ^∗∗^*p* < 0.01.

Table S1 Polymorphic molecular markers used for mapping.

Table S2 Primers used for qRT-PCR.

Table S3 GO terms.

Table S4 *LOC_Os03g20380* probe used for RNA *in situ* hybridization.

Table S5 Primers used to generate transgenic constructs.
