# Supplementary figures and images for: Rice Calcineurin B-Like Protein-Interacting Protein Kinase 31 (OsCIPK31) Is Involved in the Development of Panicle Apical Spikelets
Source: Front Plant Sci. 2018 Nov 19;9:1661. doi: 10.3389/fpls.2018.01661 (PMC6262370; doi:10.3389/fpls.2018.01661)

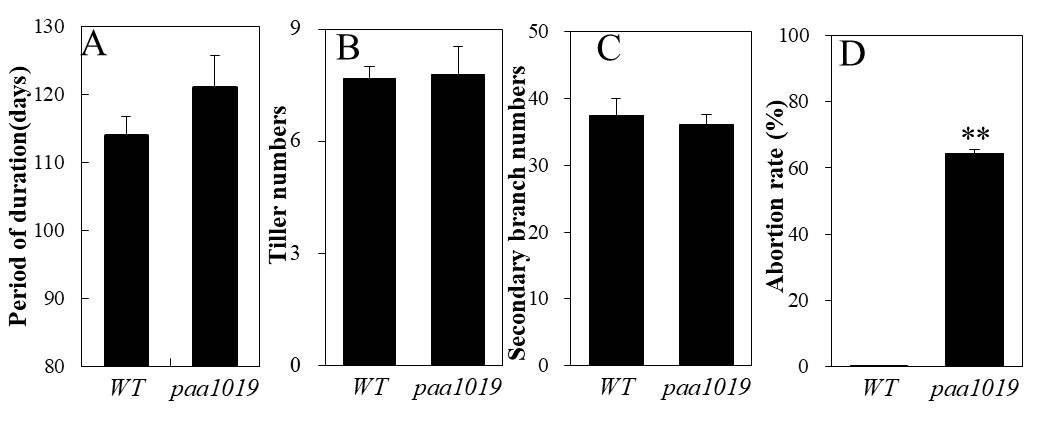

Supplement: Figure S1 — Phenotypes of the paa1019 mutant. (A-D) Agronomic traits of wild-type (WT) and paa1019 mutant plants. (A) Duration. (B) Tilling number. (C) Secondary branch number. (D) Abortion rate. Statistical analysis was performed using Student’s t-tests, ∗p < 0.05, ∗∗p < 0.01. [file Image_1.JPEG]

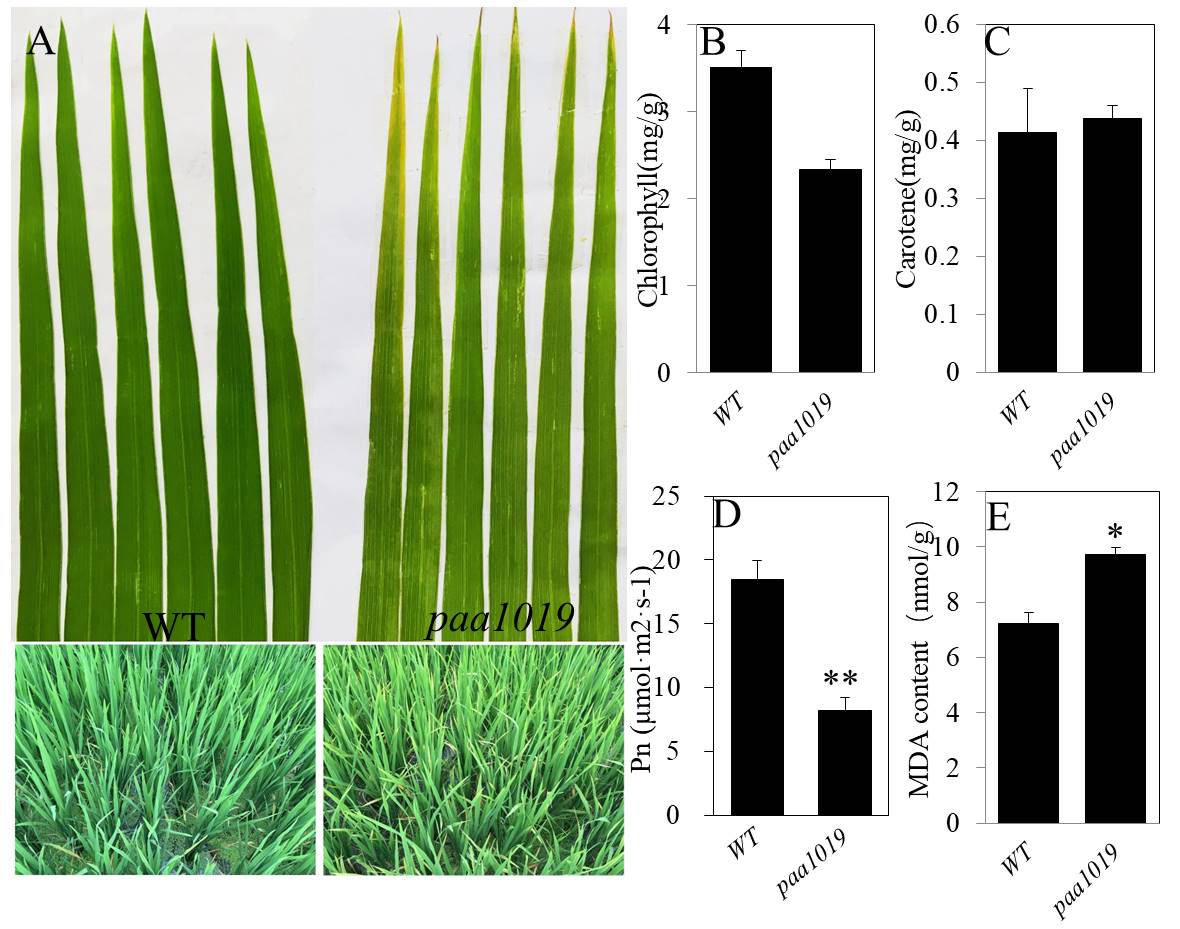

Supplement: Figure S2 — Phenotype of leaves. (A) The color of leaves in paa1019 is more yellow than in WT plants. (B-E) Content of chlorophyll (B), carotene (C), net photosynthetic rate (D), and organic acids (E). ∗p < 0.05, ∗∗p < 0.01. [file Image_2.JPEG]

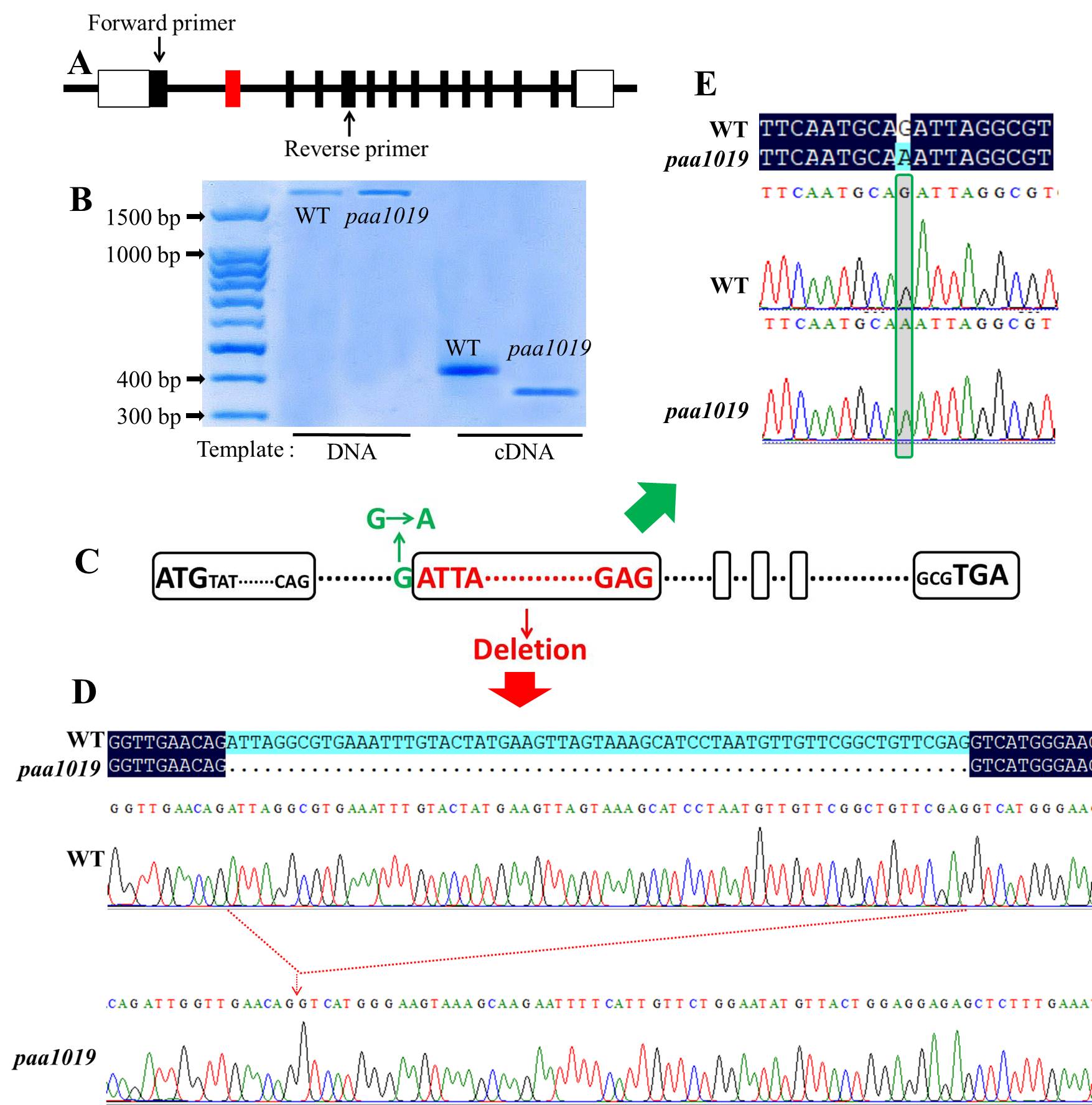

Supplement: Figure S3 — The alternative splicing of PAA1019 gene. (A) The pair of primers used for RT-PCR analysis. The forward and reverse primers were located in 1st and 5th exons, respectively. (B) RT-PCR analysis showing the presence of the abnormal size transcript of paa1019. The primers were used for amplification that is given in (A), where larger transcript was of WT (due to 2nd exon) and smaller transcript was of paa1019. The genomic DNA was used as a control. (C) The alternative splicing of PAA1019 gene in paa1019. The boxes are indicating exons, whereas the dotted lines indicate base sequences. The deleted region is highlighted in red (2nd exon), whereas base substitution is highlighted in green (that was located between the boundary of 1st intron and 2nd exon). (D) Sequencing and alignment of CDS obtained in B (WT and paa1019) showed the deletion of entire 2nd exon in paa1019. (E) Sequencing and alignment of DNA obtained in (B) revealed a base substitution from G to A in paa1019. [file Image_3.JPEG]

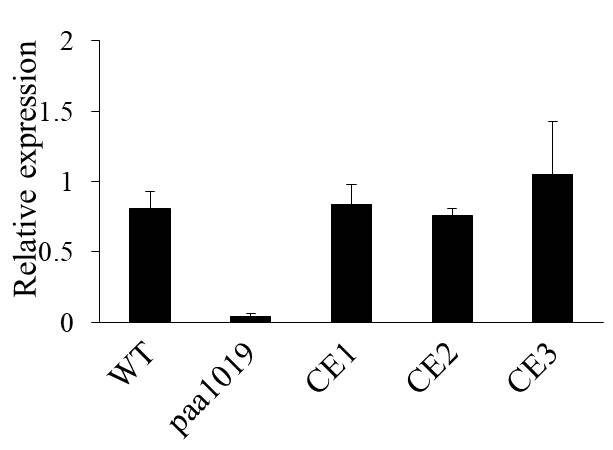

Supplement: Figure S4 — Relative expression of OsCIPK31 in WT, paa1019, and complementation lines. CE1-CE3 represent the three complementation transgenic lines. Statistical analysis was performed using Student’s t-tests. [file Image_4.JPEG]

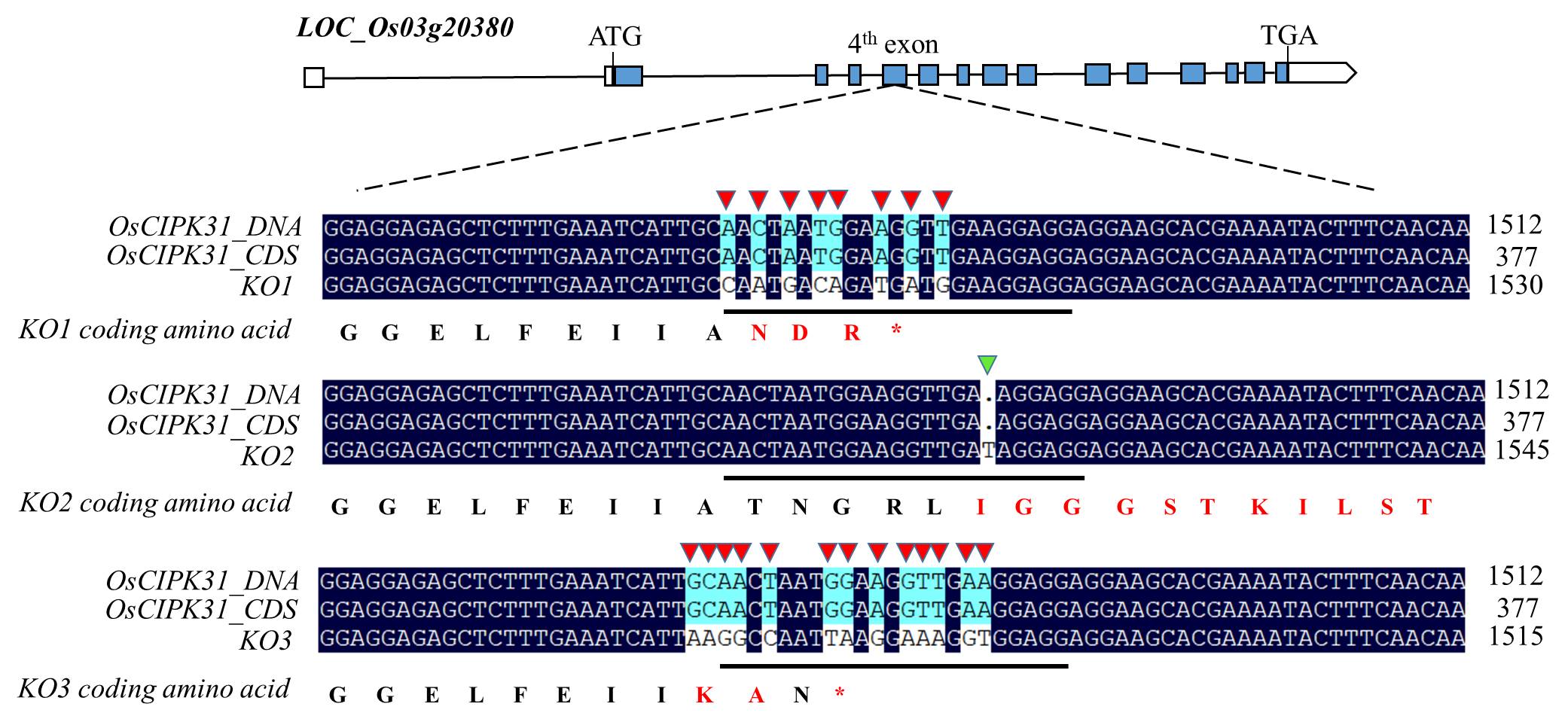

Supplement: Figure S5 — The sgRNA target sequence analysis of the knock-out lines. Different mutation at the target site in three representative knock-out lines generated by the CRISPR/Cas9 technology. The filled bars and lines are indicating exons and introns of OsCIPK31, respectively. The knock-out target region was located in the fourth exon of OsCIPK31. Mutation types of KO1 and KO3 (knock-out lines) were substitution. While the mutation type of KO2 was insertion. These mutations led to the premature termination of translation in three lines (the terminator codon is not shown in KO2 sequence). The black line indicates sgRNA target sequences. The red and green triangles are indicating the substitutive and inserted sites, respectively. OsCIPK31_DNA, OsCIPK31_CDS, and KO1/2/3 are indicating OsCIPK31 genomic, CDS, and three knock-out transgenic lines cDNA sequences, respectively. KO1/2/3 coding amino acid is indicating the corresponding amino acid sequences, where red color is depicting changed amino acid and ∗ to a termination codon. [file Image_5.JPEG]

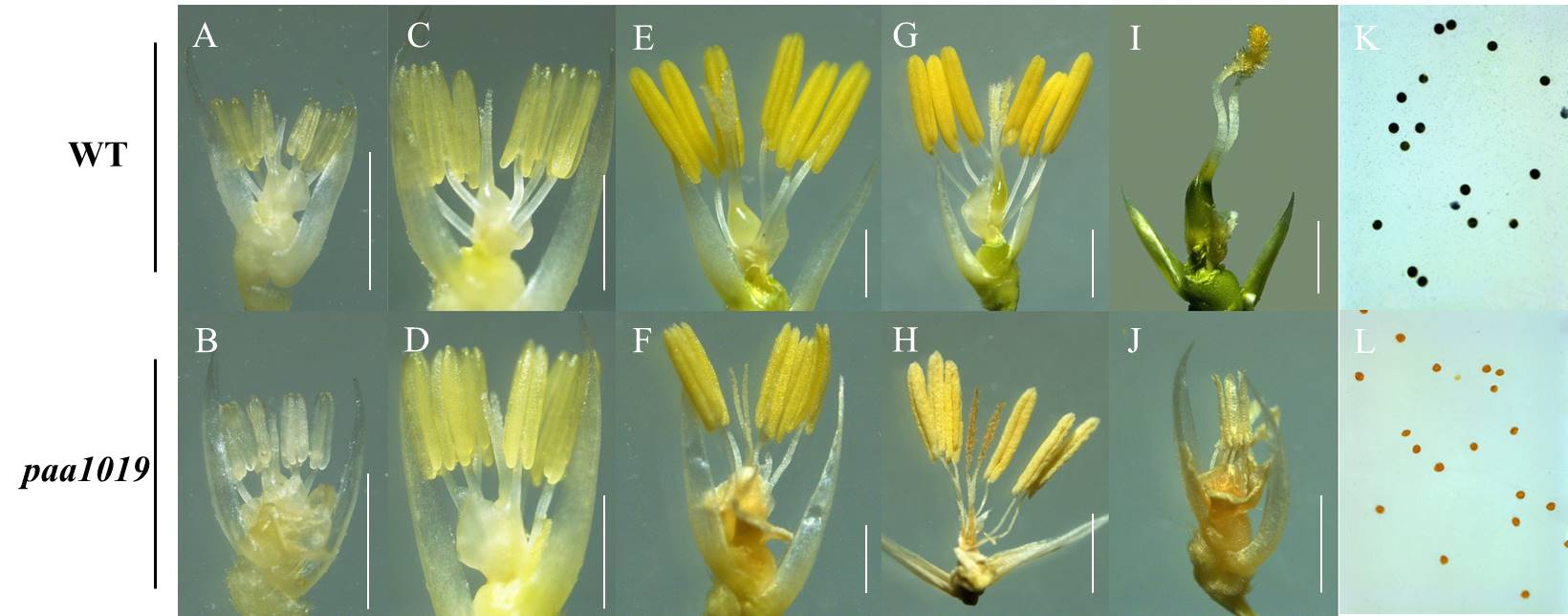

Supplement: Figure S6 — Development of flowers is arrested in the degraded spikelets of paa1019 plants. (A–J) Phenotype of flower development observed at different growth stages. (A,B) = 20 DBF; (C,D) = 15 DBF; (E,F) = 10 DBF; (G,H) = 5 DBF; (I,J) = flowering stage; (K-L) KI staining of pollen. DBF indicated days before flowering. Scale bar = 0.2 cm (A-J). [file Image_6.JPEG]

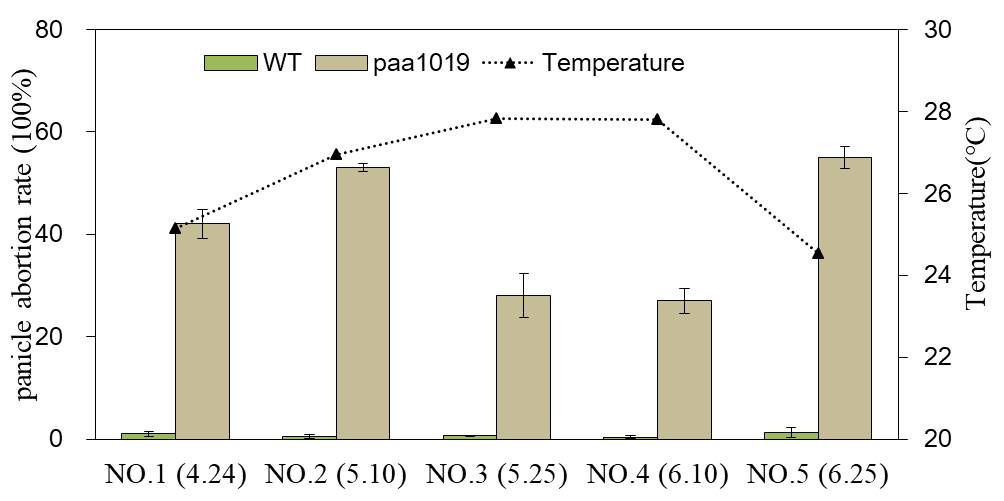

Supplement: Figure S7 — The abortion rate is influenced by the environmental temperature. [file Image_7.JPEG]

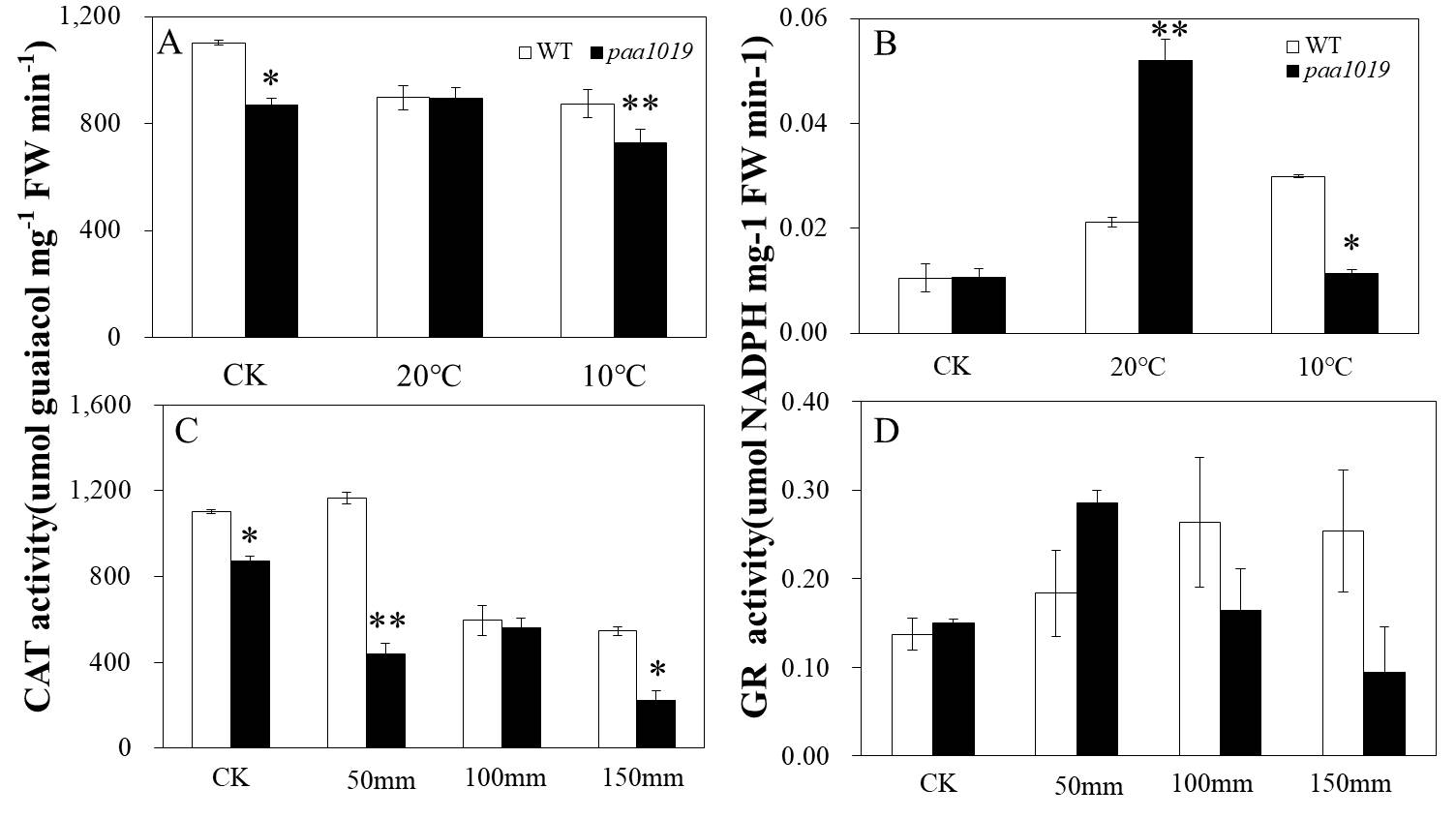

Supplement: Figure S8 — Activity of CAT and GR enzymes following different treatments. (A,B) Activity of CAT and GR enzymes following the cold treatments; (C,D) activity of CAT and GR enzymes following the salt treatments. ∗p < 0.05, ∗∗p < 0.01. [file Image_8.JPEG]

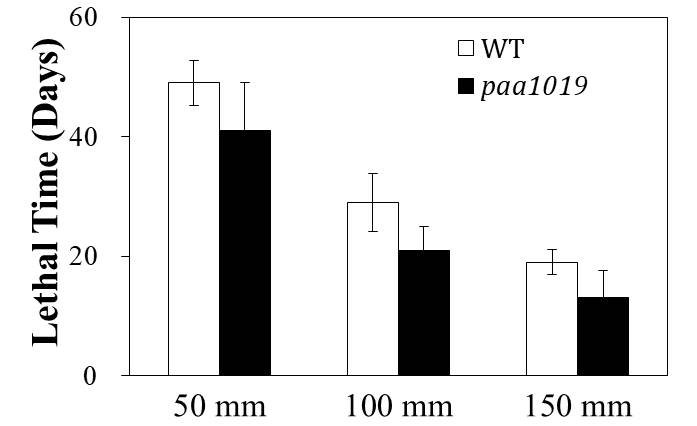

Supplement: Figure S9 — The lethal duration following different treatments. [file Image_9.JPEG]

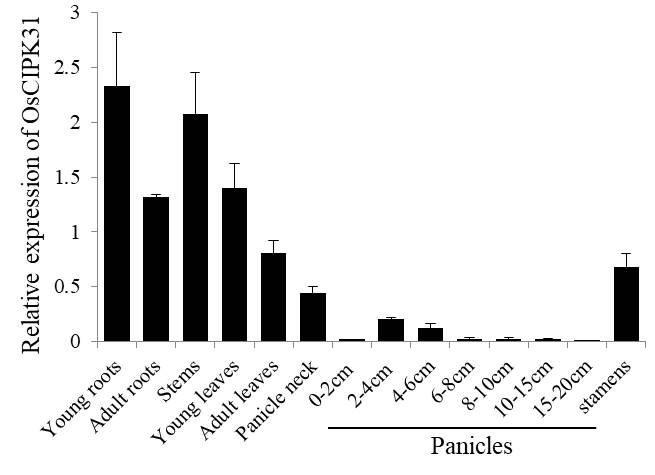

Supplement: Figure S10 — Relative expression of OsCIPK31 in wild-type. Tissues including young roots, young leaves, 0–20 cm panicles (40–70 days after germination) and stems, panicle neck, adult roots, adult leaves, stamens (80 days after germination). Rice ACTIN was used as an internal control. Data are presented as mean ± SE (n = 3). [file Image_10.JPEG]

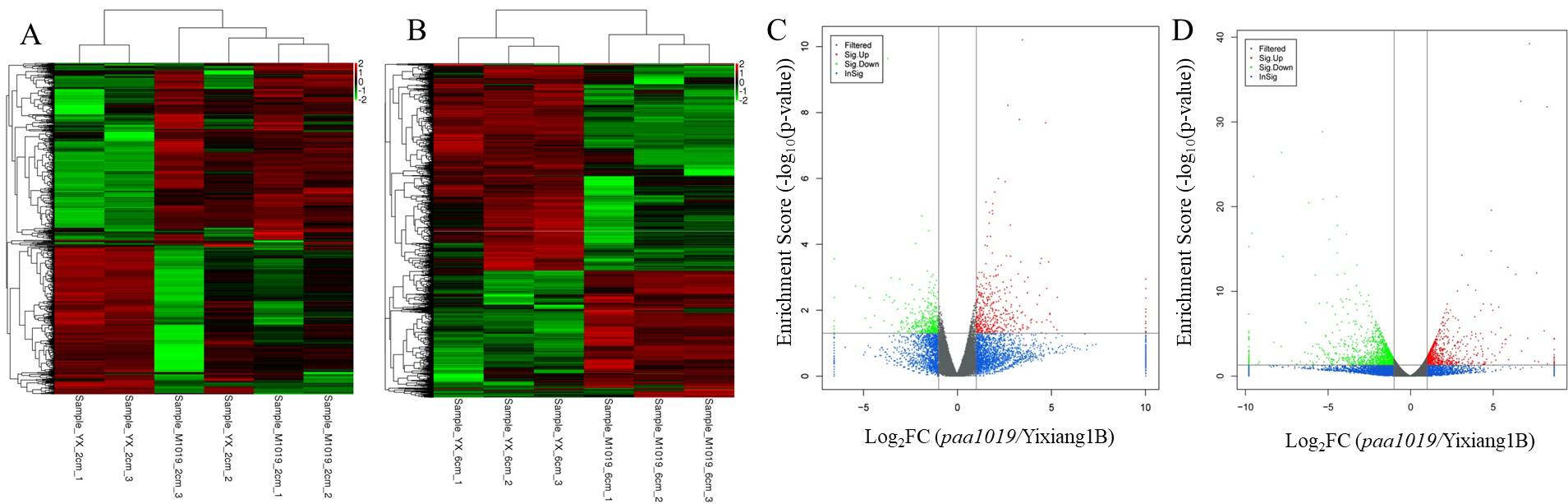

Supplement: Figure S11 — Differentially expressed genes (DEGs) between WT and paa1019 plants. (A,B) Heatmap showing the expression patterns of significantly dysregulated genes for WT-2 cm vs. paa1019-2 cm (A) and WT-6 cm vs. paa1019-6 cm (B) groups. According to DEGs analysis, significantly dysregulated genes displayed a fold change (FC) ≥ 2 and a p-value ≤ 0.05. (C,D) Volcano plot of DEGs in paa1019-2 cm (C) and paa1019-6 cm (D) relative to controls. DEGs were selected based on a false discovery rate (FDR) < 0.05 and a |log2 fold change| > 1.0. The x-axis shows the fold change in gene expression between paa1019-2 cm (C) and paa1019-6 cm (D) relative to controls, and the y-axis shows the –log10 (FDR). Blue splashes indicate genes not exhibiting significant differences in expression. Red splashes indicate significantly upregulated genes. Green splashes indicate significantly downregulated genes. Gray splashes indicate unfiltered genes. [file Image_11.JPEG]

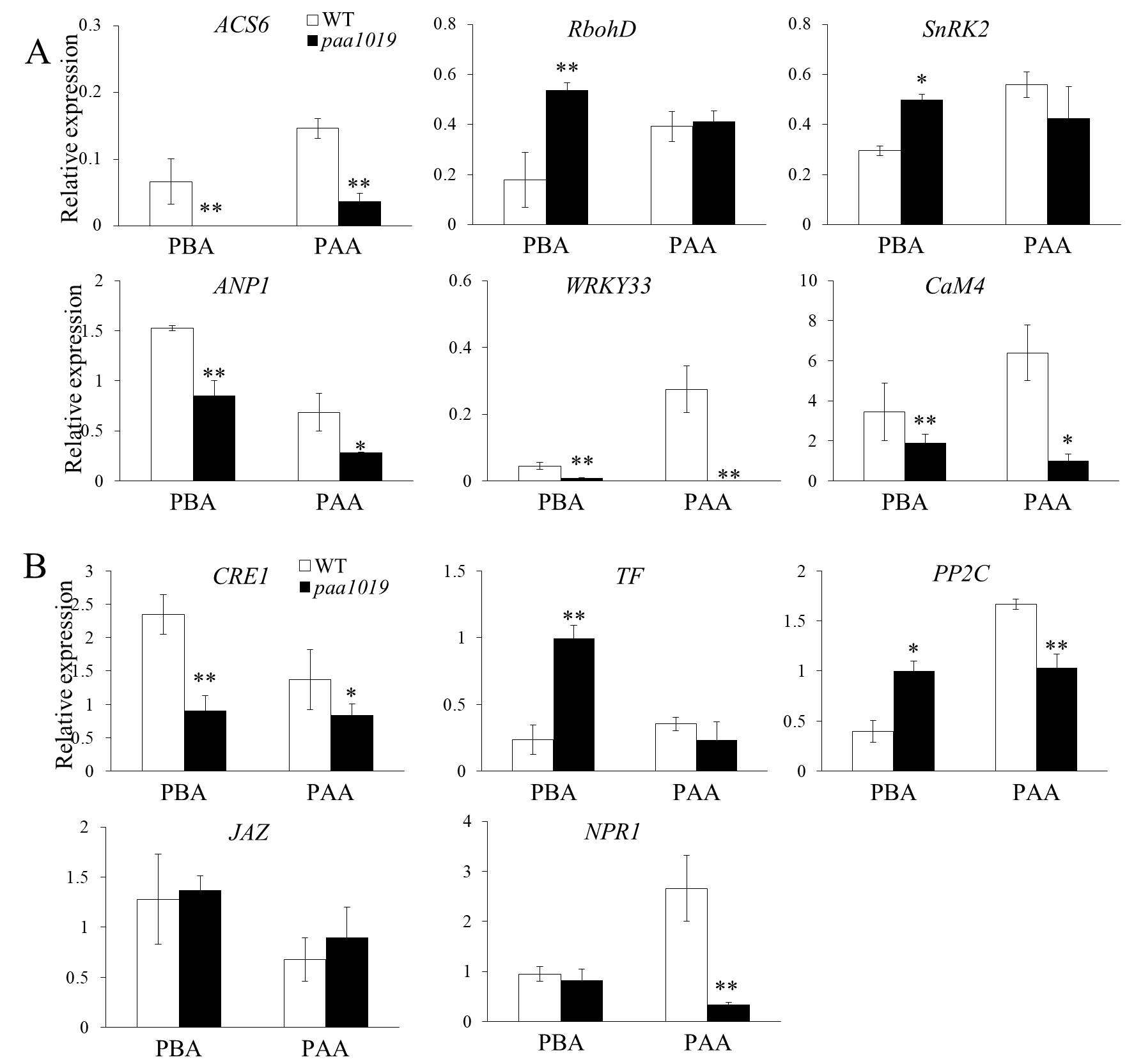

Supplement: Figure S12 — Relative expression of genes related to MAPK signaling pathway and phytohormone signal transduction. (A) Relative expression of genes related to MAPK signaling pathway. (B) Relative expression of genes related to phytohormone signal transduction. Rice ACTIN was used as an internal control. Data are presented as mean ± SE (n = 3). ∗p < 0.05, ∗∗p < 0.01. [file Image_12.JPEG]
