# Supplementary material for: Rice Calcineurin B-Like Protein-Interacting Protein Kinase 31 (OsCIPK31) Is Involved in the Development of Panicle Apical Spikelets
Source: Front Plant Sci. 2018 Nov 19;9:1661. doi: 10.3389/fpls.2018.01661 (PMC6262370; doi:10.3389/fpls.2018.01661)
Supplement: Table S1 — Polymorphic molecular markers used for mapping. [file Table_1.DOCX]

**Table S1 Polymorphic molecular markers used for mapping**

| Markers | Forward primer (5’→3’) | Reverse primer (5’→3’) |
| --- | --- | --- |
| RM251 | GAATGGCAATGGCGCTAG | ATGCGGTTCAAGATTCGATC |
| Os3-46.6 | ACATCGAGTTTAATTGGCAT | AGAGAGAAAAGGTAGGTGGG |
| Os3-46.9 | AGACAGCCAGACAAAAACAT | ACTAGGCTACTGCGACCAT |
| Os3-47.7 | AGTTGAAGCGTAAAGTCTGC | TCCAGAATTTTTCATAACCG |
| Os3-48.5 | TTCATCTGGCAAAGAGAGAT | TGTTCTTATGCTGGTTCCTT |
| Os3-48.8 | GTGGGCAATGCTAGAAAAT | GGATTATGCAAAGTCTGAGC |
| Os3-50.8 | TTTGGAGTTAGTAGTGGGCT | CGAGTTCTTCCTGATCTGTT |
| RM5748 | CAGTTGGCAATTGTCACGAG | TCGAACATATCCAAGCCTCC |
| Os3-65.4 | CTTTAAGTATGGCATATAGTTTTG | GCAAAATGAAATAACTACTCCC |
