# Supplementary material for: Rice Calcineurin B-Like Protein-Interacting Protein Kinase 31 (OsCIPK31) Is Involved in the Development of Panicle Apical Spikelets
Source: Front Plant Sci. 2018 Nov 19;9:1661. doi: 10.3389/fpls.2018.01661 (PMC6262370; doi:10.3389/fpls.2018.01661)
Supplement: Table S2 — Primers used for qRT-PCR. [file Table_2.DOCX]

**Table S2 Primers used for qRT-PCR**

| Markers | Forward primer (5’→3’) | Reverse primer (5’→3’) |
| --- | --- | --- |
| CAT1 | CCACCGATTACCGCCACA | GCCTCGTCGTCCATCAAGC |
| CAT2 | ATCCCACGCAACTACAGG | CAAGGTGAGGCTTTCCAT |
| CAT3 | CCGCTTCTCCACCGTCAT | ACCAGGTCCCAGTTGCCC |
| OsCOLD1 | CTACTACCACTGCTACCT | GAAGCCATAGAGGAAGAC |
| LTG1 | CAGACAACTTCCTTATGGGTT | ATAGCGTGCTGTTCCTGTG |
| OsP5CS | TGTGTACCAACGCGCTATGT | TATATGCATCCACGGCGATA |
| OsCIPK31 | TTCAGAAGCACAGATTG | CCGAACAACATTAGGAT |
| OsCIPK31- Splicing | GGTTTGCGAAGAACACTG | CCTTCACTTGCTCGGTTA |
| ACTIN | TGGCATCTCTCAGCACATTCC | TGCACAATGGATGGGTCAGA |
| BGIOSGA000101 | CTAACATGGCTGTTCTGGTTTC | GAGGTAGTGCATCCGCTTC |
| BGIOSGA011553 | ATATGGCGGTGAACTTCTACC | CTTGTATCTTCCGTTGCTTAGC |
| BGIOSGA016343 | AGTTCGACCCGGACAGGTT | GAACTCCACCTTGTCCTCGAAG |
| BGIOSGA021446 | ATACAACGAAACCAACATCGAC | GAAGATCCAAAGGTGCCAGATT |
| BGIOSGA006319 | GCCTAGTGATCCATACCTGAG | CTCCCAAAAGAGGCATCAGTAG |
| BGIOSGA034434 | GAGGTACAAGAAGGAGGAGAAG | GATCTCCTCCGTGATCTTGTG |
| BGIOSGA009300 | CTACTACTCGCCGTCGACTTA | GATTCATCTTGGCCTCAATTCC |
| PP2C | CACTCTCCACTGACCACAAG | AGTAGTCGCCTATGGATCTAGA |
| ACS6 | ATCATCTACTCGTACAACGACG | GATGAACTCCTCGTCCGAG |
| RbohD | CGCATTTTCGTCGAGAACTATT | AAGAATGCTTATGAAAGGCGTC |
| SnRK2 | AGTAACCCTCTACGTAATGCTG | GATGATTTCTGATCTCGGGGAT |
| ANP1 | TGGTAATGATTGGATCGAGCTT | GTTTGGCTAATCCTCCGAATTC |
| WRKY33 | GTCTTACTCTTACACGAGCCAG | CTGCTCAAAGAACGACATGATG |
| CaM4 | GTGTTCGACAAAGACCAGAAC | GAACTCCTCGTAGTTGATCTGG |
| CRE1 | GAGAAGGTGTGACAAAAATGCT | CAAATGTCGGATCAATGGTACC |
| TF | GAGTCATCGATGCTGACAATCG | TGCAGGACCTCGACCTCAT |
| JAZ | AGTACATGAAGGAGCACAGTG | CTTCCTTTCTTGCGTGTCTTTC |
| NPR1 | GTTCTCAGCTACCTGTACACC | AAGAATTGGCAGTATGTCCTCA |
