# Supplementary material for: Rice Calcineurin B-Like Protein-Interacting Protein Kinase 31 (OsCIPK31) Is Involved in the Development of Panicle Apical Spikelets
Source: Front Plant Sci. 2018 Nov 19;9:1661. doi: 10.3389/fpls.2018.01661 (PMC6262370; doi:10.3389/fpls.2018.01661)
Supplement: Table S3 — GO terms. [file Table_3.DOCX]

**Table S3 GO terms**

| **GO term description** | **Gene ID** |
| --- | --- |
| Oxidation-reduction process | *BGIOSGA000101, BGIOSGA011553, BGIOSGA016343, BGIOSGA021446* |
| Cell wall and membrane biogenesis | *BGIOSGA009099, BGIOSGA021541, BGIOSGA023124, BGIOSGA029706* |
| Transmembrane transport | *BGIOSGA004346, BGIOSGA009300, BGIOSGA031591* |
| Response to stress | *BGIOSGA009926, BGIOSGA034434* |
| Inflorescence development | *BGIOSGA006319* |
| Signal transduction- phosphorylation | *BGIOSGA034249* |
