# Supplementary material for: Rice Calcineurin B-Like Protein-Interacting Protein Kinase 31 (OsCIPK31) Is Involved in the Development of Panicle Apical Spikelets
Source: Front Plant Sci. 2018 Nov 19;9:1661. doi: 10.3389/fpls.2018.01661 (PMC6262370; doi:10.3389/fpls.2018.01661)
Supplement: Table S4 — LOC_Os03g20380 probe used for RNA in situ hybridization. [file Table_4.DOCX]

**Table S4. *LOC_Os03g20380* probe used for RNA *in situ* hybridization**

| Sequence (5’→3’) | Marker |
| --- | --- |
| CGCTT CACCT TTGGA GATAA TGCAG CCCTC | Digoxigenin (DIG) label at the 5’ end |
