# Supplementary material for: Rice Calcineurin B-Like Protein-Interacting Protein Kinase 31 (OsCIPK31) Is Involved in the Development of Panicle Apical Spikelets
Source: Front Plant Sci. 2018 Nov 19;9:1661. doi: 10.3389/fpls.2018.01661 (PMC6262370; doi:10.3389/fpls.2018.01661)
Supplement: Table S5 — Primers used to generate transgenic constructs. [file Table_5.DOCX]

**Table S5 Primers used to generate transgenic constructs**

| Marker | Forward primer (5’→3’) | Reverse(5’→3’) |
| --- | --- | --- |
| pBWA(V)BII-PROPAA1019:PAA1019 | CAGTCACCTGCAAAATAGACTCCATCCTCCTCTGCTCCT | CAGTCACCTGCAAAACGACCCATCCTCTCCTCCCTCCTC |
| CIPK31-CRISPER/Cas9 | CAGTGGTCTCAGGCAACTAATGGAAGGTTGAAGG | CAGTGGTCTCAAAACCCTTCAACCTTCCATTAGT |
|  | CAGTGGTCTCAGGCAACCTGAAAGTATCTGACTT | CAGTGGTCTCAAAACAAGTCAGATACTTTCAGGT |
| pBWA(V)BII-GUS | CAGTCACCTGCAAAATAGACTCCATCCTCCTCTGCTCCT | CAGTCACCTGCAAAACGACCCATCCTCTCCTCCCTCCTC |
| pMD1300-CIPK31-YFP-1 | GGATCCATGTATAGGGCTAAGAGGGCTGC | TCTAGACGCCGCGGCGCCGTTGCC |
| pMD1300-CIPK31-YFP-2 | CCCGGGGGAGCTAACAATTAACTACACTATTAACCTT | CCCGGGCAATAAATCAGGCTATGAAAAGGATT |
